# Making sense of complexity in context and implementation: the Context and Implementation of Complex Interventions (CICI) framework

**DOI:** 10.1186/s13012-017-0552-5

**Published:** 2017-02-15

**Authors:** Lisa M. Pfadenhauer, Ansgar Gerhardus, Kati Mozygemba, Kristin Bakke Lysdahl, Andrew Booth, Bjørn Hofmann, Philip Wahlster, Stephanie Polus, Jacob Burns, Louise Brereton, Eva Rehfuess

**Affiliations:** 1 Institute for Medical Informatics, Biometry and Epidemiology, LMU Munich, Munich, Germany; 2 Institute of Public Health and Nursing Research, University of Bremen, Bremen, Germany; 3 Health Sciences Bremen, University of Bremen, Bremen, Germany; 4 Centre for Medical Ethics, University of Oslo, Oslo, Norway; 5 School of Health and Related Research (ScHARR), University of Sheffield, Sheffield, UK

**Keywords:** Context, Implementation, Complex intervention, Concept analysis, Systematic review, Applicability, Transferability, Health technology assessment, Public health

## Abstract

**Background:**

The effectiveness of complex interventions, as well as their success in reaching relevant populations, is critically influenced by their implementation in a given context. Current conceptual frameworks often fail to address context and implementation in an integrated way and, where addressed, they tend to focus on organisational context and are mostly concerned with specific health fields. Our objective was to develop a framework to facilitate the structured and comprehensive conceptualisation and assessment of context and implementation of complex interventions.

**Methods:**

The Context and Implementation of Complex Interventions (CICI) framework was developed in an iterative manner and underwent extensive application. An initial framework based on a scoping review was tested in rapid assessments, revealing inconsistencies with respect to the underlying concepts. Thus, pragmatic utility concept analysis was undertaken to advance the concepts of context and implementation. Based on these findings, the framework was revised and applied in several systematic reviews, one health technology assessment (HTA) and one applicability assessment of very different complex interventions. Lessons learnt from these applications and from peer review were incorporated, resulting in the CICI framework.

**Results:**

The CICI framework comprises three dimensions—context, implementation and setting—which interact with one another and with the intervention dimension. Context comprises seven domains (i.e., geographical, epidemiological, socio-cultural, socio-economic, ethical, legal, political); implementation consists of five domains (i.e., implementation theory, process, strategies, agents and outcomes); setting refers to the specific physical location, in which the intervention is put into practise. The intervention and the way it is implemented in a given setting and context can occur on a micro, meso and macro level. Tools to operationalise the framework comprise a checklist, data extraction tools for qualitative and quantitative reviews and a consultation guide for applicability assessments.

**Conclusions:**

The CICI framework addresses and graphically presents context, implementation and setting in an integrated way. It aims at simplifying and structuring complexity in order to advance our understanding of whether and how interventions work. The framework can be applied in systematic reviews and HTA as well as primary research and facilitate communication among teams of researchers and with various stakeholders.

**Electronic supplementary material:**

The online version of this article (doi:10.1186/s13012-017-0552-5) contains supplementary material, which is available to authorized users.

## Background

The effectiveness of complex interventions, as well as their success in reaching all relevant target populations, is critically influenced by their implementation in a given context; indeed, effectiveness, implementation and context are inextricably linked [1, 2]. To date, however, limited information on implementation and contextual factors is reported in primary studies. Likewise, systematic reviews and health technology assessments (HTA) fail to capture context and implementation in appropriate ways, which constitutes a major barrier to appraising transferability and applicability of findings [1].

Insufficient understanding of context and implementation thus contributes to the critical gap between research and practice. Policy makers and practitioners are often challenged with making decisions in relation to the evaluation and implementation of complex interventions. Complex interventions usually comprise multiple components, which may act independently or interdependently, with the ‘active ingredient(s)’ being difficult to specify [3]. Moreover, the boundaries between what constitutes the intervention, its implementation and context are often blurred [2, 4], with interactions taking place between all three [5]. These complex interventions challenge current approaches to the conceptualisation and assessment of the intervention and the way it is implemented in context. For example, a low emission zone to reduce particulate matter air pollution and related health impacts comprises several necessary and interacting components, i.e., regulatory components (e.g., the passing of a law and its enforcement), infrastructure components (e.g., signposting) and educational components (e.g., information through mass media and other channels). A low emission zone is not implemented within one particular organisation or sector but in entire cities, such as Munich [6, 7] or London [8, 9], often requiring cooperation across multiple sectors (e.g., transport, urban development, environment, health). Relevant contextual factors include geographical aspects (e.g., existing public transport infrastructure) and socio-economic aspects (e.g., disposable income for purchasing a newer car). Importantly, this intervention is not taking place in isolation, but its impacts on particulate matter air pollution and health may be compromised or enhanced by other ongoing interventions implemented nationally (e.g., a national guideline regulating industry emissions) or locally (e.g., incentives to promote walking and cycling). Understanding whether such an intervention really makes a difference requires a conceptualisation of the intervention within the system and an appropriate assessment of context and implementation.

Numerous frameworks, models and theories for assessing implementation have been developed, together with a smaller number to assess context. In 2015, Nilsen provided a taxonomy to differentiate between categories of theories, models and frameworks in order to facilitate the appropriate selection and application of these theoretical approaches [10]. This taxonomy distinguishes between three overarching aims: describing and/or guiding the process of translating research into practise (*process models*); understanding and/or explaining what influences implementation outcomes (*determinant frameworks*, *classic theories*, *implementation theories*) and approaches facilitating the evaluation of an implementation effort (*evaluation frameworks*) [10]. Widely cited approaches, such as the Reach Effectiveness Adoption Implementation Maintenance (RE-AIM) framework or the PRECEDE-PROCEED framework [11] can thus be considered evaluation frameworks, whereas the Stetler Model or the Quality Implementation Framework [12] can be classified as process models. In contrast, the Promoting Action on Research Implementation in Health Services (PARiHS) framework [13], the Consolidated Framework for Implementation Research (CFIR) [14] or the Conceptual Model by Greenhalgh [15] represent determinant frameworks. According to Nilsen, classic theories, such as the Theory of Diffusion of Innovations [16], various social cognitive and social network or organisational theories, have been developed in psychology, sociology or organisational sciences with a realm that goes beyond implementation but can usefully explain selected aspects of implementation [10]. On the other hand, various *implementation theories*, such as the Implementation Climate Theory [17] or the Absorptive Capacity Theory [18], are directly concerned with implementation and regard behaviours that accompany or facilitate the implementation of evidence on an individual or community level [19–26]. These theories have been developed within the comparatively new field of implementation sciences, either de novo or by modifying existing theories [10].

Most existing frameworks, models and theories are primarily concerned with implementation, while context plays a minor role. The two aspects are rarely assessed in an integrated way, although selected frameworks have attempted to do so (e.g., [14, 27]). Importantly, uses of the terms ‘context’ and ‘implementation’ and their meanings vary widely in the health literature, lacking consensual definitions and descriptions as well as clearly delineated boundaries. Context is often used synonymously with setting and environment [13, 28], embracing static (e.g., physical environment) and dynamic aspects (e.g., relationships, networks) as well as the theory underpinning the intervention and its implementation [29]. Implementation is considered to be a process, a constellation of processes, efforts or the means or methods of fitting, assimilating or putting into use an intervention—either evidence-based or theory-based—in an organisation or a setting [14, 30, 31]. Moreover, many of these frameworks, theories and models are specific rather than generic, e.g., focussing only on organisational context, and therefore do not lend themselves to application across diverse health interventions. Furthermore, few of these frameworks appear suitable for use within systematic reviews and HTAs. Indeed, many of these frameworks, models and theories are relatively abstract in nature and lack guidance on how to operationalise them in practise.

Our objective was, therefore, to develop a framework to facilitate structured and comprehensive conceptualisation and assessment of context and implementation of complex health interventions. We constructed the Context and Implementation of Complex Interventions (CICI) framework for use in systematic reviews and HTAs. However, given that primary research, evidence synthesis and evidence-based policy and practise are inextricably linked [32], the framework is likely to be equally relevant for primary research.

## Methods

**Fig. 1:**
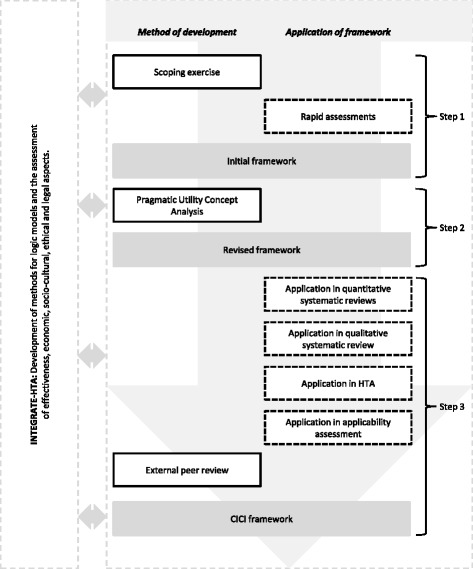
Development and application of CICI framework

The CICI framework was developed as part of the EU-funded project Integrated health technology assessment for the evaluation of complex technologies (INTEGRATE-HTA) (www.integrate-hta.eu). The overall aim of the project was to develop concepts and methods for HTA to enable a patient-centred, integrated assessment of the effectiveness, the economic, social, cultural, legal, and ethical aspects of complex health technologies, which take context and implementation into account. The CICI framework was developed in a three-step process, each involving a degree of overlap and iteration (Fig. 1).

### Step 1

In the first step, we undertook purposive literature searches for models, theories and frameworks concerned with context and/or implementation. We critically examined the publications identified in this way and, using our understanding of complex interventions within complex systems, we built an initial framework. In parallel, methods for logic models [33] as well as methods to undertake an assessment of effectiveness, economic, socio-cultural, ethical and legal aspects were developed within INTEGRATE-HTA. Starting in step 1 and continuing, we aimed to come to agreement across the project regarding the domains of the CICI framework as well as the aspects covered by each domain, so that all methods could be used in a coherent and complementary fashion (Fig. 1). As a research team, we applied the initial framework within three rapid assessments of complex interventions (i.e., improved household stoves and fuels for developing countries, specialist palliative care and e-learning interventions to increase evidence-based healthcare competencies in healthcare professionals). These rapid assessments explored whether the broad principles of the framework might apply across very different types of health interventions. The rapid assessments unveiled inconsistencies in the interpretation of the terms context and implementation and the characteristics assigned to each of these among members of the research team.

### Step 2

Consequently, we examined the conceptual maturity of both concepts (i.e., context and implementation). A mature concept is well defined and clearly described and has delineated boundaries as well as documented pre-conditions and outcomes [34]. To explore and advance conceptual maturity, we chose pragmatic utility (PU) concept analysis [34–36]. PU concept analysis comprises three major steps, i.e., selection of literature, organisation and structuring of the selected literature and asking analytical questions of the literature. In step 1, we conducted systematic searches to identify a comprehensive set of publications that describe relevant models, theories and frameworks of context and implementation. For this purpose, we conducted two separate searches, a standard systematic search for context in EMBASE and MEDLINE and an innovative forward tracking search for implementation in Google Scholar. The latter was based on the landmark review by Damschroder et al. (2009) and identified any publication citing either Damschroder et al. (2009) or the individual models, theories or frameworks included therein. This resulted in considerably improved efficiency of our searches without a significant loss of information [37]. In step 2, we structured the literature according to the field of application, the field in which the concepts were originally developed, definitions, characteristics as well as contained model(s) (e.g., Consolidated Framework for Advancing Implementation Research (CFIR)) whenever an extension or refinement of an existing framework was undertaken. Finally, in step 3, we inductively and deductively developed questions that were asked of the literature. These questions included, for example, the interactions between the concepts of context, implementation and the implementation agents, the influence of time and the conceptualisation of an implementation success. These questions were answered in order to reveal potential inconsistencies in the conceptualisation of terms. Both concepts (context and implementation) were also analysed in terms of their conceptual maturity, comprising definitions, characteristics, described pre-conditions and outcomes, and boundaries. More details on the concept analysis are published elsewhere [37]. Based on the findings of the concept analysis, we revised the initial framework.

### Step 3

The revised framework underwent extensive application. As part of the INTEGRATE-HTA project, a demonstration HTA of reinforced home-based palliative care (rHBPC) (i.e., home care with an additional element of carer support) was undertaken. The same revised framework was applied by different researchers, both within and outside of INTEGRATE-HTA. It was used (i) as part of the system-based logic model used in scoping the demonstration HTA (JB, AR, LB, LP, PW, AG, ER, KBL, KM and others) [38]; (ii) as the so called best-fit framework [39] in a qualitative systematic review (LP, AB, ER, LB and others) [38]; (iii) as the basis for a data extraction tool in a systematic review of effectiveness (JB, SP, LP, ER and others) [40–42]; and (iv) as the structure for an applicability assessment (SP, LP, AG, ER and others) (Polus et al., submitted manuscript), with most of these described in more detail below.

For the application of the revised framework in *quantitative systematic reviews*, we developed a tool for inclusion within the respective data extraction forms, with questions corresponding to the domains of the framework. This data extraction tool was used within a systematic review assessing the effectiveness of home-based palliative care undertaken as part of INTEGRATE-HTA (Burns et al., submitted manuscript), as well as a review on the effectiveness of interventions to reduce lead in consumer products and drinking water [42] and a Cochrane review of interventions to reduce ambient air pollution [40].

In a *qualitative systematic review* of contextual enablers and barriers to the implementation of home-based palliative care [38], we used the revised framework as a best-fit framework, meaning that findings emerging deductively from the data were structured according to the framework; where findings did not fit, the framework was modified inductively. To facilitate this process, we developed a further data extraction tool based on domains of the framework, with three questions to represent each of the domains.

We also applied the revised framework within an *applicability assessment*, which served to examine whether the findings of the demonstration HTA would be applicable in distinct decision-making settings. To identify contextual and implementation factors affecting applicability, we conducted semi-structured consultations with palliative care experts from three European countries. The domains of the revised framework provided the structure for the consultation guide, which included an open question on each domain (Polus et al., submitted manuscript).

Drawing on recognised approaches for examining applicability, coherence, completeness, usefulness and ease of application [43, 44], we compiled seven criteria (Table 1) to guide our appraisal of whether the framework could actually deliver on what we had set out to achieve, i.e., to facilitate the structured and comprehensive conceptualisation and assessment of context and implementation of complex health interventions. We collected feedback on the above from all of the researchers who had participated in the various applications, either verbally or in writing. At the same time, the framework was externally peer-reviewed by three experts with intentions to apply the framework. These experts, characterised by different methodological backgrounds and activities across different fields (medical and social policy), provided detailed written feedback; in one case, this was pursued further through an in-depth discussion.

**Table 1:** Criteria for appraising applicability of the CICI framework

Internal coherence and completeness of framework	
Coherence	Is the framework internally coherent and consistent? Are the definitions clear?
Completeness	Is the framework comprehensive?
Theory advancement and development	
Theory	To what extent does the framework facilitate the advancement or development of theories?
Compatibility	To what extent is the framework compatible with other theories, frameworks or models?
Relationships	To what extent does the framework allow for the assessment and appraisal of relationships between its components?
Applicability of framework	
Adaptation and applicability	To what extent can the framework be applied and/or adapted to different interventions?
Flexibility	To what extent can the framework be applied in systematic reviews and HTAs employing different methods?
Capability	To what extent does the framework capture complexity?
User-friendliness of framework	
Feasibility of application	Can the framework be applied easily?

**Fig. 2:**
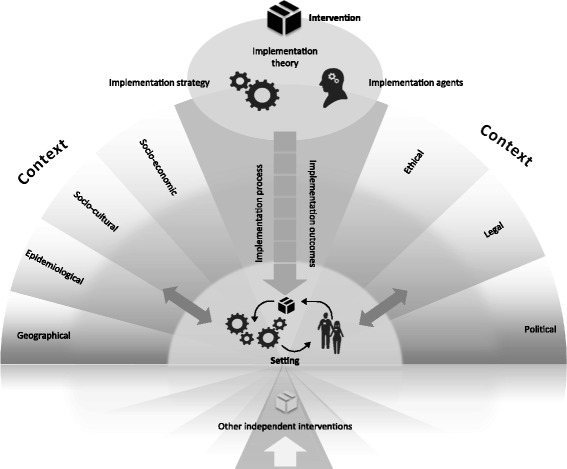
CICI framework. The context and implementation of complex interventions (CICI) framework comprises the three dimensions context, implementation and setting. The context comprises the seven domains: geographical, epidemiological, socio-cultural, socio-economic, ethical, legal, political context. Implementation consists of implementation theory, implementation process, implementation strategies, implementation agents and implementation outcomes. In the setting, the intervention and its implementation interact with the context. The *shading* of the *semicircles* illustrates the micro, meso and macro levels, on which implementation, context and setting can occur. Apart from the intervention of interest, the context and the way the intervention of interest is implemented may be advanced or compromised by other interventions occurring independently but targeting the same setting and population

Based on lessons learnt from the multiple applications as well as the detailed feedback from the external peer reviewers, we modified the framework, resulting in the CICI framework presented here (Fig. 2).

We then developed a worked example by applying the framework to an exemplary complex intervention included in the abovementioned Cochrane review of interventions to reduce ambient air pollution: the Air Pollution Act on the Marketing, Sale and Distribution of Coal, was initially introduced in Ireland in 1987 and enacted in the city of Dublin from 1990. Since we were also aiming to demonstrate the added value of the CICI framework as compared to other established and widely used frameworks, we also applied the CFIR [14] and the PARiHS framework [27] to the same intervention. In populating the three frameworks, we started with the primary study included with our Cochrane review [45] and then conducted specific searches of the grey literature to identify relevant information in newspaper articles, government documents, city council reports as well as lobbyism reports.

## Results

### Initial framework (step 1)

The initial framework comprised two dimensions—context and implementation—with eight domains (i.e., locational, geographical, epidemiological, socio-cultural, socio-economic, ethical, legal and political) and four domains (i.e., provider, organisation and structure, funding and policy), respectively. The intervention, informed by theory, lies at the heart of the CICI framework with its reach and effectiveness being affected by context and implementation.

### Towards the revised framework (step 2)

Based on the systematic searches, we included 35 publications developing, proposing or describing theories, models or conceptual frameworks for implementation (see Additional file 1) and 17 publications doing the same for context (see Additional file 2) [37]. The concept of implementation is situated within an advancing and well-connected field of implementation science. The concept of context, on the other hand, is less studied and applied in diverse ways across different fields, among them implementation science. Setting—a term that is sometimes used synonymously with context—emerged as an additional and distinct concept of interest. Our searches had not been designed to capture all relevant aspects, which precluded a formal concept analysis. The definitions of context, setting and implementation as advanced by concept analysis are presented in Table 2. The concept analysis led to a clear conceptual distinction between context and setting as well as a more comprehensive formulation of characteristics for each of the domains of context and implementation. These were incorporated in the revised framework.

**Table 2:** Definitions of context, implementation and setting based on Pragmatic Utility concept analysis [37]

Context	*Context* reflects a set of characteristics and circumstances that consist of active and unique factors, within which the implementation is embedded. As such, context is not a backdrop for implementation, but interacts, influences, modifies and facilitates or constrains the intervention and its implementation. Context is usually considered in relation to an intervention, with which it actively interacts. It is an overarching concept, comprising not only a physical location but also roles, interactions and relationships at multiple levels.
Implementation	*Implementation* is an actively planned and deliberately initiated effort with the intention to bring a given intervention into policy and practise within a particular setting. These actions are undertaken by agents who either actively promote the use of the intervention or adopt the newly appraised practises. Usually, a structured implementation process consisting of specific implementation strategies is used and underpinned by an implementation theory.
Setting	*Setting* refers to the specific physical location, in which the intervention is put into practise and interacts with context and implementation.

### Towards the CICI framework (step 3)

The revised framework was applied to different complex interventions using various methodological approaches (i.e., HTA, quantitative systematic reviews, qualitative systematic review, applicability assessment). We mapped feedback received from the researchers applying the revised framework in HTA, quantitative and qualitative reviews as well as an applicability assessment together with our own experiences against the criteria in Table 1. Overall, as summarised in Table 3, the revised framework showed good internal coherence and completeness, applicability and user-friendliness and was found to contribute to theory advancement and development. The test cases did, however, also reveal that selected aspects under specific domains of context and implementation had been missing.

**Table 3:** Main findings obtained from appraising the applicability of the framework

Internal coherence and completeness of framework	
Coherence	In terms of *coherence*, the framework was appraised as largely consistent; significant overlaps between the context and implementation dimensions were noted. In the qualitative review, issues arose in relation to the attribution of text extracts to specific domains (e.g., provider vs. organisational domain). Also, data extractors were not comfortable with our original definition of the ethical domain.
Completeness	In relation to *completeness*, researchers considered the framework complete at the level of the domains but not with respect to the aspects covered. Consequently, missing aspects (e.g., health care system) were added. In the applicability assessment, the framework partly missed the complexity and adaptability of the intervention [14]. In particular, the concept of reinforced home-based palliative care, as defined for the HTA, did not fit the German context, where reinforcement (e.g., carer support) is integrated in every palliative care construct available.
Theory advancement and development	
Theory	While its generic version does not display relationships, the framework allows for their assessment when applied to a complex intervention. The framework was also considered helpful in guiding the formulation of questions about these links, which in return inspires the advancement of theory regarding interactions between domains as well as context and implementation dimensions.
Compatibility	The framework was assessed as *compatible* with other methods developed and applied within INTEGRATE-HTA (i.e., assessments of ethical or socio-cultural issues, logic model and the INTEGRATE-HTA model) as a consequence of their development taking place side-by-side. As the framework builds on a systematic review of previously published conceptual frameworks, theories and models of context and implementation, it can also be considered compatible with the literature.
Relationships	In line with the tradition of previous implementation frameworks, the application of the revised CICI framework in quantitative reviews does not make *relationships* between dimensions or domains explicit. On the other hand, in the qualitative systematic review, the framework facilitated the assessment of relationships through data extracts being attributed to several domains, for example, ‘access to healthcare’ emerged as a relevant aspect under three context domains (geographical, political, ethical and socio-economic). Moreover, the graphical display arising from its use highlights how dimensions and domains interact within a complex system.
Applicability of framework	
Adaptation and applicability	A certain degree of *adaptation* with regard to the domains to be considered in relation to a specific intervention is clearly a strength of the framework. However, to avoid ‘cherry-picking,’ the decision of which domains to consider should be a structured and transparent process. The successful application of the framework to very different types of health interventions suggests that the criterion *applicability* is met. While studies looking at policy and environmental interventions reported more details on the macro context, studies evaluating educational, psychoeducational, psychological, social as well as spiritual interventions reported more details on the meso level relating to context or setting.
Flexibility	Generally, the framework has proven to be methodologically *flexible* due to its good applicability in quantitative and qualitative systematic reviews and the primary qualitative, quantitative or mixed-method studies included in these, although data extraction was sometimes limited by poor reporting. The framework also showed flexibility in relation to the demonstration HTA as a whole and the applicability assessment.
Capability	The high granularity of the framework proved *capable* of facilitating extraction of—often scarcely—reported information on context and implementation and supported the structuring of this information. It therefore helps to review all sources of complexity in relation to a given intervention and to examine the interactions between them.
User-friendliness of framework	
Feasibility of application	The framework was considered feasible to apply across all the different applications; ease of use was ensured through clear use of terminology, step-by-step guidance and the provision of ready-to-use data extraction tools. In quantitative reviews, extracting data on context and implementation imposes an additional workload on researchers. The additional time required is limited, where extraction is only performed on the included study; it may be substantial if further sources (e.g., qualitative studies or process evaluations cited in the included study) are also consulted.

In general, the peer reviewers felt that the guidance was useful and comprehensive but questioned whether the framework would be taken up in practise, given the additional workload required. They also expressed some concern with respect to the definition of the implementation domains. Related to this, they issued a collective call for a clearer distinction between the context and implementation dimensions and greater elaboration of definitions of individual domains. As a consequence, the implementation dimension was significantly revised. In contrast, the context dimension remained largely unchanged apart from the locational domain being subsumed within a new “setting” dimension.

The CICI framework comprises three dimensions*—context*, *implementation* and *setting*—which are described in detail in the following section. The *context* dimension comprises seven domains, i.e., geographical, epidemiological, socio-cultural, socio-economic, ethical, legal and political. The *implementation* dimension comprises five domains, i.e., implementation theory, implementation process, implementation strategies as well as implementation agents and outcomes. *Setting* refers to the specific physical location, in which the intervention is put into practise. In the setting dimension, context, implementation and the intervention, which are described according to intervention theory, design and delivery characteristics [46], interact with one another and often co-evolve over time. Clearly, the intervention itself is critical. This dimension and its three domains intervention theory, intervention design (including components and execution) as well as intervention delivery are described in Table 4; they have been defined and described in detail elsewhere [46]. The CICI framework fundamentally builds on and is applied alongside this conceptualisation of the intervention but focuses on an operationalisation of context, setting and implementation.

**Table 4:** The intervention dimension and its domains

Intervention		
Dimension	Definition	Aspects
Intervention theory	The body of implicit or explicit ideas about how an intervention works [2, 100] and includes the overall aims of the intervention [46]	-Theory of intervention -Goal of intervention
Intervention design	The description of the “What?” of the intervention [46]	-Components of intervention oTechnology and infrastructure oEducation oPolicy and regulations -Execution of intervention oTiming and duration oDose and intensity
Intervention delivery	The description of the “How?”, “Who?” and “Where?” of the intervention [46]	-Delivery mechanisms -Delivery agents

### Macro, meso or micro levels

Depending on the nature of the intervention, the interactions between intervention, implementation and context in a given setting can occur at a macro level (e.g., policies or regulations across a whole health system or country), meso level (e.g., introduction of new treatment guidelines in a specific hospital or of sanitation systems in a specific village) or micro level (e.g., promotion of health-protective behaviours among individuals or families). Interventions are typically implemented simultaneously within different settings and at multiple levels. It is, however, not necessarily useful or meaningful to conduct an analysis at all levels, and the relevant level will depend on the assessed intervention and the system, in which it exists. The *macro* level refers to everything surrounding a community or organisation [37]; this can include the regional, national or international environment. The *meso* level refers to a community or organisation [47]*.* A community is defined by its function (e.g., employer, religious entity), geography (e.g., village, neighbourhood), shared interests or characteristics (e.g., ethnicity, culture) or a combination of these [48, 49], with members sharing a sense of identity or connection [50, 51]. An organisation is defined by its structure and size [52–55], organisational culture [14, 52, 56–62] and climate [14, 63], networks and relationships [64–66]. All of these community or organisational characteristics jointly influence implementation climate [14, 15], system readiness for change [14, 17, 67] and capacity for change [15, 62, 68, 69] and thus the chances of an intervention being implemented successfully. The *micro* level refers to the level of direct action, i.e., where the intervention is delivered by a specialised palliative care team at the patient’s home, in which a room has to be remodelled in order to deliver physiotherapy to a patient.

Apart from the intervention of direct interest, the context and the way the intervention of interest is implemented may be advanced or compromised by other interventions occurring independently. For example, an organisation promoting water flushing before tap water consumption to avoid exposure to lead in drinking water in a specific community (meso level) can be facilitated by an ongoing national campaign on the risks of lead contamination of drinking water (macro level). These other interventions are likely to influence the reach and effectiveness of the intervention under investigation in a specific setting.

### Context domains

The geographical context refers to the broader physical environment, landscapes and resources, both natural and transformed by humans (e.g., infrastructure), available in a given setting. The supply of rHBPC, for example, might be hindered by the availability of services for geographically isolated potential recipients of palliative care.

The epidemiological context refers to the distribution of diseases or conditions, the attributable burden of disease, as well as determinants of needs in populations [5], including demographics [70, 71]. Psychosocial and physical needs of lay caregivers of palliative care patients as well as the needs of palliative care patients themselves would fall into this category.

The socio-cultural context comprises explicit and implicit behaviour patterns, including their embodiment in symbols and artefacts; the essential core of culture consists of historically derived and selected ideas and values that are shared among members of a group [72]. It not only refers to the conditions in which people are born, grow, live, work and age but also embraces the social roles a human being takes on as a family member, community member or citizen and the relationships inherent to these roles. Constructs such as knowledge, beliefs, conceptions, customs, institutions and any other capabilities and habits acquired by a group are included in this domain [73]. An example could be families and communities that fulfil specific roles in the provision of home-based palliative care (HBPC). The wife of a palliative care patient could feel obliged to care for her husband due to social expectations; being an informal carer in HBPC may lead to loss of employment or income.

The socio-economic context comprises the social and economic resources of a community and the access of a population to these resources [14, 74]. This could, for example, comprise the potential loss of income among lay caregivers of palliative care patients as they enter the caregiver role.

The ethical context comprises reflections of morality, which encompasses norms, rules, standards of conduct and principles that guide the decisions and behaviour of individuals and institutions [75]. Ethical, socio-cultural and legal aspects are strongly interrelated [76–78]. For example, ensuring autonomy and valid consent for patients receiving palliative care at home with their informal carers (both are care receivers) can be challenging given their different needs, preferences, legal position, and socio-cultural perspectives.

The political context focuses on the distribution of power, assets and interests within a population, as well as the range of organisations involved, their interests and the formal and informal rules that govern interactions between them [79]. The domain also comprises the health care system and its accessibility (e.g., delivery of services, leadership and governance, health information, human resources and financing). For example, the increasing political pressure to ensure equitable access to palliative care for those in rural areas has led to greater interest in telemedicine [80]. In this case, interactions with the geographical and the socio-cultural domains were observed.

The legal context is concerned with the rules and regulations that have been established to protect a population’s rights and societal interests [75]. Formally, these have to be passed by a competent legislative body like a parliament. Legal norms can mostly be enforced with order and compulsion, which distinguishes them from ethical and social norms [81]. A legal issue arising in HBPC on the micro level is, for example, the sharing of information with relatives who wish to be informed about the medical condition of the palliative care patient. This might contradict the legal framework, in which care is delivered as well as socio-cultural and ethical norms of information sharing.

### Implementation domains

#### Implementation theory

An implementation theory attempts to explain the causal mechanisms of implementation [10]; it is therefore analogous to a programme theory, which attempts to explain the causal mechanisms linking an intervention and its outcomes. An implementation theory formalises how change needs to be executed in order for the implementation effort to be successful [70, 82, 83] and underpins both the implementation process and implementation strategies [37].

#### Implementation process

The implementation process refers to the social processes, through which interventions are operationalised in an organisation or community [84]. It contains the tactics and methods used by change leaders [62, 82]. The implementation process is an active, multistage, iterative and dynamic process that does not usually occur in a linear fashion [82]. At specific points, corrections, refinements or expansions [14, 82] are undertaken by implementation agents [85] in order to successfully implement an intervention.

The first stage is characterised by the exploration of organisational needs, intervention-organisational fit as well as capacity and readiness assessment in a given setting [12, 86]. Once the decision to adopt an intervention has been made [31, 53], structural changes can be undertaken in the setting in order to facilitate the implementation effort [82]. This requires thorough planning and preparation, a stage during which specific implementation strategies, the engagement of implementation agents as well as the implementation process itself are planned [14, 53, 82]. This is followed by initial implementation [54, 62, 82, 87]. At this stage, staff should be educated and information disseminated; interventions can be pilot-tested and consequently adapted [87]. Full implementation begins, when the intervention becomes integrated into practise at all levels and for all implementation agents and intervention recipients [54, 82]. At this stage, processes and procedures supporting the intervention execution are in place, and the system, although never completely stable, has largely been recalibrated to accommodate and support the intervention [82]. The stage of evaluation and reflection aims to assess the process as well as the outcomes with reference to the intended goals, and to learn from the implementation for the setting in question, as well as for future implementation efforts in other settings [12, 14, 31, 62]. Evaluation and reflection often occur relatively late in the implementation process; however, ideally, this starts from the very beginning. The final stage relates to sustainment of a successfully implemented intervention and refers to the continued use of the intervention in the organisation or community [53, 54, 86].

#### Implementation strategies

The implementation process consists of specific implementation strategies, which encompass all methods and means to ensure the adoption and sustainment of interventions [37]. Drawing on the implementation theory and epistemological considerations, implementation strategies comprise a set of activities that are chosen and tailored to fit a specific context [52, 88] or to create such a context [52]. These may change over time. Implementation strategies can contain multiple components and as such may be considered an intervention in its own right. Implementation strategies should be named, preferably according to the literature in order to identify similar efforts [83], and should be described in terms of their components [83]. Furthermore, implementation strategies can be described in terms of their theory, the implementation agents, the concrete action, the action target, their temporality, their dose as well as the implementation outcome(s) affected [83].

#### Implementation agents

Implementation agents comprise all individuals and organisations engaged with (i) deciding to implement a given intervention (e.g., funders, administrators), (ii) implementing this intervention (e.g., providers, advocates, physicians, nurses) or (iii) being the target or otherwise affected by an intervention (e.g., patients and their families, consumers) [37]. These implementation agents can be located inside or outside (e.g., external change agents) of the organisation, in or through which an intervention is implemented. These individuals have particular personality attributes, skills, knowledge, beliefs as well as attitudes that exert their influence on the implementation of an intervention. The success of implementation is highly dependent on the buy-in of individuals who become key stakeholders in both the intervention and the implementation effort [89]. Thus, stakeholders should be carefully involved with the planning, execution and evaluation of the implementation effort. An implementation effort can be promoted and facilitated by single implementation leaders [14, 15] and/or implementation teams [82].

Individual implementation agents, or teams that are actively involved in funding, administering or implementing an intervention, are usually embedded in an organisation or community that critically influences their attitudes and behaviours (see description of meso level above). This organisational or community level determines the overall implementation climate and capacity for change and thus the ability of individual implementation agents to contribute to an implementation effort. As such, it plays a significant role in implementing an intervention successfully. This is especially true, where one organisation or group within an organisation is selected among several alternatives, or where a new organisation is created to facilitate implementation.

#### Implementation outcomes

An implementation outcome is the result or implication of the implementation effort and forms part of good monitoring and evaluation practises. Important implementation outcomes are adoption, uptake, acceptability, implementation cost, penetration, sustainability [14, 89] and dissemination to other contexts [14].

### Operationalisation of the CICI framework

#### CICI framework tools

To facilitate the pragmatic application of the CICI framework, we developed a generic checklist (Table 5). This checklist can be modified with respect to a specific intervention and the level(s), at which its implementation is to be assessed (i.e., macro, meso, micro); it can and should also be adapted and refined towards specific uses in primary research, evidence synthesis and evidence-based policy and practise. The checklist comprises questions regarding which factors of a respective dimension (i.e., context, implementation, setting) exert their influence, and how this influence affects implementation success and, ultimately, intervention effectiveness. A table describing the aspects within the domains can be found in Additional file 3.

**Table 5:** CICI framework generic checklist

Intervention	
Intervention characteristics	• Which intervention characteristics interact with the setting, the context and the implementation? • How do these intervention characteristics interact with the setting, the context and the implementation?
Context	
*Depending on the intervention of interest, all or a subset of the seven domains of context should be reflected upon, i.e., geographical, epidemiological, socio-cultural, socio-economic, ethical, legal and political context.*	
Context	• Which aspects of the context interact with the implementation of the intervention? • How do these aspects of the context interact with the intervention? • How do these aspects of the context interact with implementation?
Implementation	
Implementation theory	• Which theoretical underpinning guides the implementation? • How does this theory interact with the setting and the context? • How does this theory interact with the intervention?
Implementation process	• Which stages of the implementation process are passed through during implementation? • How does the implementation process interact with the setting and the context? • How does the implementation process interact with the intervention?
Implementation strategy	• Which implementation strategies are employed during implementation? • How do these implementation strategies interact with the setting and the context? • How do these implementation strategies interact with the intervention?
Implementation agents	• Which implementation agents are involved in the implementation effort? • How do these implementation agents interact with the setting and the context? • How do these implementation agents interact with the intervention?
Implementation outcomes	•Which implementation outcomes are reported? • How do these implementation outcomes interact with the intervention outcomes?
Setting	
Setting	• Which aspects of the setting interact with the intervention? •How does the setting interact with the intervention? •How does the setting interact with the context? • How does the setting interact with the implementation?

When used in a systematic review or HTA, the CICI framework can be operationalised through the use of a data extraction tool. Generic data extraction tools for qualitative and quantitative systematic reviews, as applied in several systematic reviews, are available (see Additional files 4 and 5). The expert consultation guide is also accessible for use (see Additional file 6).

#### Lessons learnt from a worked example

The worked example of the CICI framework, available in Additional file 7 and also including a simple logic model of the intervention, demonstrates that the framework can be readily applied to a complex public health intervention that operates across multiple settings and engages multiple implementation agents across different organisations and sectors. Issues identified in scientific articles and grey literature documents could easily be categorised under relevant domains, and the CICI framework also facilitated explicit consideration of potential interactions occurring between domains and dimensions and across various levels. These interactions were listed in our tabular presentation of findings and can thus be made transparent. It should be noted, however, that a more in-depth analysis of interactions would necessitate a thorough textual presentation.

The assessment of context shed light on factors that influence implementation success and, ultimately, the effectiveness of the intervention in reducing ambient particulate matter air pollution and associated mortality; for example, the lack of a strong coal lobby and collaboration across different ministries positively influenced implementation, whereas historically grown preferences for the use of solid fuels for heating and concerns about the loss of jobs in the coal industry initially presented challenges.

The CICI framework accommodated with ease the consideration that the intervention was implemented in more than one setting: the Irish government itself, the local authority that is obliged to adopt and enforce the regulation, the coal vendor that needs to register with the Environmental Protection Agency as well as the single household within the enforcement zone that reacts by changing to a different fuel or heating system. All of these settings involve different implementation agents, require different implementation strategies and allow the assessment of different implementation outcomes.

In contrast, we had difficulties with applying the CFIR and PARiHS to our intervention of choice, as both assume that those delivering and those receiving the intervention (e.g., doctors, nurses) are nested within the organisation implementing the change (e.g., hospitals, health centres); consequently, the detailed assessment of context exclusively relates to this organisational context. Instead, for public health/complex interventions, there is often an organisational or even sectoral disconnection between the implementing organisation(s) and those receiving the intervention, and context is concerned with the society at large. Consequently, as we applied the CFIR and PARiHS to the Air Pollution Act, many relevant aspects of context and implementation were not captured at all, while a substantial proportion of the CFIR and PARiHS domains remained unpopulated. Importantly, from our application of the CICI framework, we learnt that context as a versatile, dynamic construct evolves over time, as the intervention unfolds and as implementation strategies change, e.g., the issuing of additional taxes or grants. A further difficulty related to the terminology used, where terms used by the CFIR and PARIHS frameworks (e.g., patients) are grounded in a clinical and/or health services perspective and only partially applicable to public health interventions.

## Discussion

Numerous frameworks, theories and models assessing implementation and, to a lesser extent, context have been published over the past decades. Few of these frameworks address implementation and context in an integrated fashion, and very few offer pragmatic guidance and worked examples of how to apply the framework, theory or model in practise. To our knowledge, the CICI framework is the first attempt to provide guidance for assessing setting, context and implementation of complex interventions in a comprehensive manner, for integrating the views of different disciplines (economics, ethics, sociology, law) and for adapting a concept of context that is embedded in a broad public health perspective rather than a narrower organisational perspective. Through systematic literature searches and concept analysis, the CICI framework builds on previous knowledge and presents a strong theoretical basis; through empirical applications across several distinct complex health interventions, it has proven its value across a range of different methodological approaches (e.g., qualitative and quantitative systematic reviews, primary qualitative research). It can serve both as a *determinant framework* that seeks to conceptualise, describe and understand the multiple influences on implementation outcomes, and as an *evaluation framework* that clarifies the context, setting and implementation aspects to be assessed when examining implementation success or lack thereof. The CICI framework includes step-by-step pragmatic guidance, a generic checklist to be adapted for different purposes and data extraction tools as well as a graphical representation to facilitate its application in practise.

### Theoretical underpinnings and scope

Previously published theories, frameworks or models, as identified from our systematic searches are based on different theoretical underpinnings, although these are often not made explicit. We are aware of at least two other concept analyses of context [90]. McCormack and colleagues looked at context as part of the Promoting Action on Research Implementation in Health Services (PARiHS) framework [37]; Squires and colleagues have not yet published their concept analysis [90]. To our knowledge, no concept analysis of implementation has been undertaken to date.

Our concept analysis, which was a critical building block for the development of the CICI framework, attempted to capture all relevant aspects of context, implementation and setting, keeping a complex interventions perspective in mind. In terms of previous frameworks, models or theories, Damschroder’s Consolidated Framework for advancing Implementation Research, Greenhalgh’s landmark review, Roger’s Diffusion of Innovation Theory, and Klein and Sorra’s Theory of Innovation [17] and Ajzen’s Theory of Planned Behaviour were particularly influential in the development of the CICI framework. Integrating insights gained in previously published frameworks, theories or models, it adds a macro perspective that seeks to capture all aspects of complex interventions. In moving beyond the organisational context, the framework is particularly suitable for public health and other interventions that are assessed or analysed on a macro level or on several levels concurrently. Previously published approaches primarily focus on implementation, although the details of implementation theory, implementation strategy or a comprehensive implementation process are often neglected (e.g., [14, 53, 84, 85]). Context often plays a subordinate role; where it is considered explicitly, it is usually restricted to an organisational context [27, 44, 91]. As demonstrated in the worked example, for complex interventions context extends much beyond the organisational context due to the number of settings, in which the intervention is delivered, as well as the number of implementation agents and strategies active across different sectors. Moreover, the ethical, socio-economic or epidemiological context, concepts that are regularly used in HTA and are known to have a considerable impact on the uptake, reach and effectiveness of an intervention, are rarely considered. The CICI framework highlights this breadth and depth of the influence of context from a societal perspective and shows that context can act at one or several different levels (micro, meso and macro), thereby adding granularity and flexibility to the assessment of a complex intervention. Lastly, the CICI framework pays tribute to competing interventions that might have a considerable impact on the uptake, reach and effectiveness of the intervention being investigated.

Over the last few decades, considerable work has been undertaken to elaborate and define elements of organisational context as well as behaviours that influence an implementation effort (REFS). This work is referenced throughout the relevant domains, and, where applicable to the intervention in question, we would advise users of the CICI framework to thoroughly examine the constructs and sub-constructs elaborated in these other frameworks. For example, respective subdomains within the implementation strategy domain of the CICI framework align with the work of Proctor and colleagues [83]. In this way, the CICI framework can serve as a comprehensive starting point for examining context and implementation; where useful, granularity within specific domains can be added by bringing in insights from previously published frameworks.

As stated repeatedly throughout this paper, an intervention and its implementation interact in multiple ways within any given setting and context, with these interactions taking place at multiple levels. Going further, it may be argued that implementation and context are constitutive parts of the intervention itself. Therefore, any structural separation of these dimensions may be considered somewhat artificial but, at the same time, is necessary in order to facilitate a structured description and a feasible assessment. As noted before, it is hard to fully account for the complex and interdependent relationships between the various domains of context and implementation, although we found that these can be tracked and made transparent using the tools presented in this paper. It is important to note that these interact in a complex adaptive system, where significant changes at a micro, meso or macro level can become manifest over time [92]. We encourage researchers conducting systematic reviews or HTA to use the CICI framework in conjunction with a system-based logic model [46], since both were developed in a coherent manner as two complementary tools. The logic model is designed to facilitate a detailed description of the theory, design and delivery of an intervention and to take into account the interactions between the intervention, its implementation and the surrounding system; the latter is the focus of the CICI framework. Worked examples of combined applications of both tools are the systematic reviews on HBPC, interventions to reduce lead in consumer products and drinking water as well as interventions to reduce ambient air pollution [40–42].

### Need for detailed assessment and reporting

The success in populating the dimensions and domains of the CICI framework with evidence critically depends on detailed assessment and reporting of information in primary studies. In our various applications of the framework as well as in the worked example, a significant problem was that primary studies reported little detail on context and implementation; this was less problematic when using primary qualitative or mixed-method studies. Unfortunately, our experience confirms what is known from the literature: reporting of information on context and implementation of health interventions is consistently poor [93]. While many of the widely used reporting guidelines, such as STROBE, SQUIRE, TREND and the extension of CONSORT for Pragmatic Trials, acknowledge context, most only require researchers to report on the setting. More specific reporting guidelines, such as the *Reporting guidelines for implementation and operational research*, the revised *Criteria for Reporting the Development and Evaluation of Complex Interventions in healthcare* (CReDECI 2) [94], the *REporting of studies Conducted using Observational Routinely collected Data statement* (RECORD) [95] and *Developing Standards for Reporting Phase IV Implementation studies* (StaRI) [96] provide helpful guidance on how to report implementation. Additional information regarding context can moreover be found in process evaluations that attest to the evolving, adaptive dynamics of context [97].

### Methodological strengths and limitations

The combination of systematic searches to identify existing frameworks, theories and models of context and implementation and formal methods to derive definitions and detailed characteristics of the relevant concepts provides a strong theoretical basis for the CICI framework and constitutes one of its major strengths. Some limitations pertaining to the searches are described elsewhere, for example the lack of explicit searches in databases of management and organisational studies [37]. It must also be noted that the setting dimension is less well conceptualised, mostly because our search strategy was designed to yield insights into the concepts of context and implementation. The realisation that setting, which plays a critical role in many fields, in particular in health promotion, should be treated as a separate dimension only became apparent in step 3. While the literature tends to conceptualise setting as having physical boundaries [98], this may fall short of acknowledging developments during the last decade (e.g., telemedicine, Internet- or smart phone-based interventions). Importantly, we did not employ any formal method of consensus building, such as a Delphi approach. The iterative development of the framework through regular interactions with other methodological work packages across the INTEGRATE-HTA project and external peer review of the framework cannot compensate entirely for this but demonstrates good general agreement with the principles of the framework from a broad group of experts across many different disciplines.

Another considerable strength of the CICI framework is that it underwent extensive testing across different types of interventions and using a range of methodological approaches (i.e., HTA, effectiveness reviews, qualitative review and applicability assessment). While the CICI framework was developed as a generic framework with broad applicability and flexibility, it is not intended to be a straitjacket: depending on the intervention being examined and the scope of the assessment, the CICI framework can focus on selected aspects or domains of the context, implementation and setting dimensions and thus can be tailored to the needs imposed by the intervention or research question. In summary, according to the feedback received, the framework performed well against seven pre-specified criteria. A limitation is, however, that the latest version of the framework has not yet been fully tested. We are in the process of applying the CICI framework in several of our own primary research and evidence synthesis projects and will critically examine its performance. In addition, we would greatly value feedback from others applying the framework and suggestions on how to make it more useful.

## Conclusions

As the complexities that emerge in relation to implementing effective health interventions become ever more apparent, it becomes increasingly important to undertake the systematic conceptualisation and assessment of context and implementation. Complexity is not only inherent in the intervention but also a consequence of interactions between the intervention and its implementation in context. The CICI framework constitutes one way of structuring this complexity in order to advance our understanding of whether and how interventions work, while keeping in mind that it is impossible to look for and discover everything [99]. When used in conjunction with a logic model, the CICI framework can help researchers and research organisations that undertake systematic reviews and HTAs, such as Cochrane and the European network for HTA (EUnetHTA), make sense of complexity and develop, prioritise and hopefully answer some of the many questions that arise when implementing complex interventions. Operationalised as a checklist and/or graphical representation, the framework can help researchers and research organisations to communicate with the policy and practise audiences they intend to reach. Finally, while developed primarily for evidence synthesis, the usefulness of the framework depends on the evidence available. Therefore, we hope that the scientific community will support adoption of the CICI framework for primary research, where it may be used for conceptualising interventions in complex systems taking the influence of setting, context and implementation on intervention reach and effectiveness into account.

## Additional files

Additional file 1: Publications included into concept analysis of context. (DOC 79 kb)
Additional file 2: Publications included into concept analysis of implementation. (DOC 93 kb)
Additional file 3: Dimensions, Domains and Constructs of Context, Implementation and Setting. (DOC 484 kb)
Additional file 4: Data Extraction Tool for Quantitative Systematic Reviews. (DOC 54 kb)
Additional file 5: Data Extraction Tool for Qualitative Systematic Reviews. (DOC 55 kb)
Additional file 6: Expert Consultation Guide. (DOC 57 kb)
Additional file 7: Worked example of three frameworks (CICI, CFIR, PARIHS). (DOCX 143 kb)

## Abbreviations

CFIR: Consolidated framework for implementation research
CICI: Context and Implementation of Complex Interventions
CONSORT: Consolidated Standard of Reporting Trials
CReDECI 2: Criteria for Reporting the Development and Evaluation of Complex Interventions in healthcare
EUnetHTA: European network for health technology assessment
HBPC: Home-based palliative care
HTA: Health technology assessment
PARiHS: Promoting Action on Research Implementation in Health Services
PRISM: Practical, Robust Implementation and Sustainability Model
PU: Pragmatic utility
RE-AIM: Reach Effectiveness Adoption Implementation Maintenance framework
RECORD: REporting of studies Conducted using Observational Routinely collected health Data
rHBPC: Reinforced home-based palliative care
StaRI: Standards for Reporting Phase IV Implementation studies
STROBE: STrengthening the Reporting of OBservational studies in Epidemiology
TREND: Transparent Reporting of Evaluations with Nonrandomized Design
WHO: World Health Organisation
