# Supplementary material for: Making sense of complexity in context and implementation: the Context and Implementation of Complex Interventions (CICI) framework
Source: Implement Sci. 2017 Feb 15;12:21. doi: 10.1186/s13012-017-0552-5 (PMC5312531; doi:10.1186/s13012-017-0552-5)
Supplement: Additional file 1: — Publications included into concept analysis of context. (DOC 79 kb) [file 13012_2017_552_MOESM1_ESM.doc]

# ***Additional File 1: Publications included into concept analysis of context***

| Anderson LM, Scrimshaw SC, Fullilove MT, Fielding JE, Task Force on Community Preventive (2003) The Community Guide's model for linking the social environment to health. Am J Prev Med 24: 12-20. |
| --- |
| Bergstrom A, Peterson S, Namusoko S, Waiswa P, Wallin L (2012) Knowledge translation in Uganda: a qualitative study of Ugandan midwives' and managers' perceived relevance of the sub-elements of the context cornerstone in the PARIHS framework. Implement Sci 3: 117 |
| Burchett H, Umoquit M, Dobrow M (2011) How do we know when research from one setting can be useful in another? A review of external validity, applicability and transferability frameworks. Journal of health services research & policy 16: 238-244. |
| Chaudoir SR, Dugan AG, Barr CH S.R. Chaudoir, A.G. Dugan, C.H.I. Barr (2013) Measuring factors affecting implementation of health innovations: a systematic review of structural, organizational, provider, patient, and innovation level measures. Implement Sci 8: 22. |
| Damschroder LJ, Aron DC, Keith RE, Kirsh SR, Alexander JA, et al. (2009) Fostering implementation of health services research findings into practice: a consolidated framework for advancing implementation science. Implement Sci 4: 50. |
| Estabrooks CA, Squires JE, Cummings GG, Birdsell JM, Norton (2009) Development and assessment of the Alberta Context Tool. BMC health services research 9. |
| Frohlich KL, Potvin L, Chabot P, Corin E (2002) A theoretical and empirical analysis of context: neighbourhoods, smoking and youth. Soc Sci Med 54: 1401-1417. |
| Kayser-Jones J(1992) Culture, environment, and restraints: a conceptual model for research and practice. Journal of gerontological nursing 18: 13-20. |
| McCormack B, Kitson A, Harvey G, Rycroft-Malone J, Titchen A, Seers K.. McCormack, A. Kitson, G. Harvey, J. Rycroft-Malone, A. Titchen, et al. (2002) Getting evidence into practice: the meaning of 'context'. J Adv Nurs 38: 94-104. |
| McCormack B, McCarthy G, Wright J, Slater P, Coffey A (2009) Development and testing of the Context Assessment Index (CAI). Worldviews Evid Based Nurs 6: 27-35. |
| Mendel P, Meredith LS, Schoenbaum M, Sherbourne CD, Wells KB. (2008) Interventions in organizational and community context: a framework for building evidence on dissemination and implementation in health services research. Adm Policy Ment Health 35: 21-37. |
| Riedmann D, Jung M, Hackl WO, Stuhlinger W, van der Sijs H, et al. (2011) Development of a context model to prioritize drug safety alerts in CPOE systems. BMC Med Inform Decis Mak 11: 35. |
| Sorensen G, Emmons K, Hunt MK, Barbeau E, Goldman R, et al. (2003) Model for incorporating social context in health behavior interventions: applications for cancer prevention for working-class, multiethnic populations. Prev Med 37: 188-197. |
| SURE Collaboration (2011) 5. Identifying and addressing barriers to implementing policy options. SURE Guides for Preparing and Using Evidence-Based Policy Briefs (Version 2.0): The SURE Collaboration. |
| Tomoaia-Cotisel A, Scammon DL, Waitzman NJ, Cronholm PF, Halladay JR, et al. (2013) Context matters: the experience of 14 research teams in systematically reporting contextual factors important for practice change. Ann Fam Med 11 Suppl 1: S115-123. |
| Wells M, Williams B, Treweek S, Coyle J, Taylor J, Wells M, Williams B, Treweek S, Coyle J, Taylor J (2012) Intervention description is not enough: evidence from an in-depth multiple case study on the untold role and impact of context in randomised controlled trials of seven complex interventions. Trials 13. |
| World Health Organization (WHO); (2013) How to use the ICF: A practical manual for using the International Classification of Functioning, Disability and Health (ICF). Exposure draft for comment. Geneva: WHO. |
