# Supplementary material for: Making sense of complexity in context and implementation: the Context and Implementation of Complex Interventions (CICI) framework
Source: Implement Sci. 2017 Feb 15;12:21. doi: 10.1186/s13012-017-0552-5 (PMC5312531; doi:10.1186/s13012-017-0552-5)
Supplement: Additional file 2: — Publications included into concept analysis of implementation. (DOC 93 kb) [file 13012_2017_552_MOESM2_ESM.doc]

# ***Additional File 2:*** Publications included into concept analysis of implementation

| Aarons GA, Fettes DL, Hurlburt MS, Palinkas LA, Gunderson L, Willging CE, et al. Collaboration, Negotiation, and Coalescence for Interagency-Collaborative Teams to Scale-Up Evidence-Based Practice. Journal of Clinical Child & Adolescent Psychology: 2014;43(6):915-28 |
| --- |
| Aarons GA, Horowitz JD, Dlugosz LR, Ehrhart MG. The role of organizational processes in dissemination and implementation research. In: Brownson RC, Graham AC, Proctor EK, editors. Dissemination and Implementation Research in Health: Translating Science to Practice. Oxford: Oxford University Press; 2012. p. 128-53. |
| Aarons GA, Hurlburt M, Horwitz SM. Advancing a Conceptual Model of Evidence-Based Practice Implementation in Public Service Sectors. Administration and Policy in Mental Health and Mental Health Services Research. 2011;38(1):4-23. |
| Avgar AC, Litwin AS, Pronovost PJ. Drivers and Barriers in Health IT Adoption A Proposed Framework. Applied Clinical Informatics. 2012;3(4):488-500. |
| Beidas SR, Edmunds J, Ditty M, Watkins J, Walsh L, Marcus S, et al. Are Inner Context Factors Related to Implementation Outcomes in Cognitive-Behavioral Therapy for Youth Anxiety? Administration and Policy in Mental Health and Mental Health Services Research. 2013;41(6):788-99. |
| Cane J, O'Connor D, Michie S. Validation of the theoretical domains framework for use in behaviour change and implementation research. Implement Sci. 2012 Apr 24;7:37. |
| Chaudoir SR, Dugan AG, Barr CHI. Measuring factors affecting implementation of health innovations: a systematic review of structural, organizational, provider, patient, and innovation level measures. Implementation Science. 2013;8. |
| Damschroder LJ, Aron DC, Keith RE, Kirsh SR, Alexander JA, Lowery JC. Fostering implementation of health services research findings into practice: a consolidated framework for advancing implementation science. Implement Sci. 2009;4:50. |
| Damschroder LJ, Aron DC, Keith RE, Kirsh SR, Alexander JA, Lowery JC. Fostering implementation of health services research findings into practice: a consolidated framework for advancing implementation science. Implementation Science. 2009;4. |
| Damschroder LJ, Hagedorn HJ. A Guiding Framework and Approach for Implementation Research in Substance Use Disorders Treatment. Psychology of Addictive Behaviors. 2011;25(2):194-205. |
| Emmons KM, Weiner B, Fernandez ME, Tu S-P. Systems Antecedents for Dissemination and Implementation: A Review and Analysis of Measures. Health Education & Behavior. 2012;39(1):87-105. |
| Fixsen DL, Blase KA, Naoom SF, Wallace F. Core Implementation Components. Research on Social Work Practice. 2009;19(5):531-40. |
| Flottorp SA, Oxman AD, Krause J, Musila NR, Wensing M, Godycki-Cwirko M, et al. A checklist for identifying determinants of practice: A systematic review and synthesis of frameworks and taxonomies of factors that prevent or enable improvements in healthcare professional practice. Implementation Science. 2013; 8:35. |
| Glanz K, Bishop DB. The Role of Behavioral Science Theory in Development and Implementation of Public Health Interventions. In: Fielding JE, Brownson RC, Green LW, editors. Annual Review of Public Health, Vol 31. Annual Review of Public Health. 312010. p. 399-418. |
| Green AE, Fettes DL, Aarons GA. A Concept Mapping Approach to Guide and Understand Dissemination and Implementation. Journal of Behavioral Health Services & Research. 2012;39(4):362-73. |
| Hage E, Roo JP, van Offenbeek MAG, Boonstra A. Implementation factors and their effect on e-Health service adoption in rural communities: a systematic literature review. BMC Health Services Research. 2013;13. |
| Helfrich CD, Li Y-F, Sharp ND, Sales AE. Organizational readiness to change assessment (ORCA): Development of an instrument based on the Promoting Action on Research in Health Services (PARIHS) framework. Implementation Science. 2009; 4:38. |
| Kaplan HC, Brady PW, Dritz MC, Hooper DK, Linam WM, Froehle CM, et al. The influence of context on quality improvement success in health care: a systematic review of the literature. The Milbank Quarterly. 2010;88(4):500-59. |
| Kitson A, Powell K, Hoon E, Newbury J, Wilson A, Beilby J. Knowledge translation within a population health study: how do you do it? Implementation Science. 2013; 8:54. |
| May C. Towards a general theory of implementation. Implementation Science. 2013;8:18. |
| May CR, Mair F, Finch T, MacFarlane A, Dowrick C, Treweek S, et al. Development of a theory of implementation and integration: Normalization Process Theory. Implementation Science. 2009; 4:29 |
| Metz A, Bartley L. Active Implementation Frameworks for Program Success. Zero to One. 2012. |
| Meyers DC, Durlak JA, Wandersman A. The Quality Implementation Framework: A Synthesis of Critical Steps in the Implementation Process. American Journal of Community Psychology. 2012;50(3-4):462-80. |
| Packard T. Organizational Change: A Conceptual Framework to Advance the Evidence Base. Journal of Human Behavior in the Social Environment. 2013;23(1). |
| Palmer D, Kramlich D. An Introduction to the Multisystem Model of Knowledge Integration and Translation. Advances in Nursing Science. 2011;34(1):29-38. |
| Proctor EK, Powell BJ, McMillen JC. Implementation strategies: recommendations for specifying and reporting. Implementation Science. 2013; 8:139 |
| Rycroft-Malone J, Seers K, Chandler J, Hawkes CA, Crichton N, Allen C, et al. The role of evidence, context, and facilitation in an implementation trial: implications for the development of the PARIHS framework. Implementation Science. 2013; 8:28. |
| Simpson DD. A framework for implementing sustainable oral health promotion interventions. Journal of Public Health Dentistry. 2011;71:S84-S94. |
| Stetler CB, Damschroder LJ, Helfrich CD, Hagedorn HJ. A Guide for applying a revised version of the PARIHS framework for implementation. Implementation Science. 2011; 6:99. |
| Suter E, Deutschlander S, Lait J. Using a Complex Systems Perspective to Achieve Sustainable Health Care Practice Change. Journal of Research in Interprofessional Practice and Education. 2011;2(1):83-99. |
| Suter E, Goldman J, Martimianakis T, Chatalalsingh C, DeMatteo DJ, Reeves S. The use of systems and organizational theories in the interprofessional field: findings from a scoping review. Journal of Interprofessional Care. 2013;27(1):57-64. |
| Talsma A, McLaughlin M, Bathish M, Sirihorachai R, Kuttner R. The Quality, Implementation, and Evaluation Model: A Clinical Practice Model for Sustainable Interventions. Western Journal of Nursing Research. 2014;36(7):929-46. |
| Taxman FS, Belenko S. Conceptual Model: Evidence Based Interagency Implementation Model. In: Taxman FS, Belenko S, editors. Implementing Evidence-Based Practices in Community Corrections and Addiction Treatment. New York: Springer 2012. p. 239-74. |
| Tomoaia-Cotisel A, Scammon DL, Waitzman NJ, Cronholm PF, Halladay JR, Driscoll DL, et al. Context matters: the experience of 14 research teams in systematically reporting contextual factors important for practice change. Annals of Family MedicineAnn Fam Med. 2013;11 Suppl 1:S115-23. |
| VanDeusen Lukas C, Engle RL, Holmes SK, Parker VA, Petzel RA, Nealon Seibert M, et al. Strengthening organizations to implement evidence-based clinical practices. Health Care Management Review. 2010;35(3):235-45. |
| Weiner BJ. A theory of organizational readiness for change. Implement Sci. 2009;4:67. |
