# Supplementary material for: Making sense of complexity in context and implementation: the Context and Implementation of Complex Interventions (CICI) framework
Source: Implement Sci. 2017 Feb 15;12:21. doi: 10.1186/s13012-017-0552-5 (PMC5312531; doi:10.1186/s13012-017-0552-5)
Supplement: Additional file 3: — Dimensions, Domains and Constructs of Context, Implementation and Setting. (DOC 484 kb) [file 13012_2017_552_MOESM3_ESM.doc]

# ***Additional File 3: Dimensions, Domains and Constructs of Context, Implementation and Setting***

**Table 1**: Domains of Context

| Domain | Definition | Aspects |
| --- | --- | --- |
| Geographical | The geographical characteristics refer to the broader physical environment, landscapes and resources, both natural and transformed by humans, available at a given location. As such, it also comprises the infrastructure at a given location, which could result in geographical isolation. | Depending on the specific intervention, describing this domain may include:  **Refinement according to concept analysis:**   - geographical isolation [5]   **Initial framework as based on the scoping review:**   - Geography (e.g. altitude, desert, forest, water) - Climate (e.g. temperature, rainfall) - Human land use (e.g. degree of urbanisation, agriculture, industry) [6, 7] - Infrastructure (e.g. water and sanitation, energy, transport) [8] - Relevant changes over time (e.g. infrastructure development, crop failures) |
| Epidemiological | This domain refers to the distribution of disease/conditions, the attributable burden of disease as well as determinants of needs in human populations [9]. Therefore, it also includes demographics [5, 10].  . | Depending on the specific intervention, describing this domain may include:  **Refinement according to applications:**   - Determinants of needs of people indirectly affected by disease/condition (e.g. lay caregivers of palliative care patients cared for at home)   **Refinement according to concept analysis:**   - Determinants of needs of people directly affected by disease/condition - Burden of disease [11]   **Initial framework as based on the scoping review:**   - Demographics (life expectancy, gender, age, ethnicity, genetic factors) [5, 10] - Population density, fertility patterns, family size [7] - Incidence/prevalence and severity of disease, morbidity and mortality [11] - Spatial distribution of disease across geographical areas - Relevant changes over time (e.g. epidemics) |
| Socio-cultural | This domain comprises explicit and implicit behaviour patterns, including their embodiment in symbols and artefacts; the essential core of culture consists of historically derived and selected ideas and values that are shared among members of a group [12] It not only refers to the conditions in which people are born, grow, live, work and age but also embraces the social roles a human being takes in as a family member, community member or citizen and the relationships inherent to these roles. Constructs such as knowledge, beliefs, conceptions, customs, institutions and any other capabilities and habits acquired by a group are comprised by this domain [13] | Depending on the specific intervention, describing this domain may include:  **Refinement according to applications:**   - none   **Refinement according to concept analysis:**   - Sociodemographic profiles [11] - Psychosocial factors [1, 14] - Social and societal context [15, 16] - Structural social inequalities (e.g. Gender inequalities, caste system) [5] - Community characteristics and level of coordination/involvement with community [17]   **Initial framework as based on the scoping review:**   - Language and means of communication - Symbols, heroes, rituals [18] - Values (e.g. evil vs. good, dirty vs. clean, dangerous vs. safe, abnormal vs. normal) [18] - Beliefs (e.g. superstition, fate or destiny) - Religiosity and spirituality - Knowledge and perceptions (e.g. with respect to significance of health issue, options for resolving health issue, multiple benefits and drawbacks of technology) - Lifestyle (population’s patterns in nutrition, smoking, substance abuse) [19] - Income equality, and racial discrimination [20] - Social capital (i.e. ability of actors to secure benefits by virtue of membership in social networks and other social structures [21]) and resources available through social relationships [22] [23], specifically social networks, norms of reciprocity, and trust [24, 25]. - Social structure: Social cohesion, including relational, material, and political dimensions, information exchange, networks of support, and informal social control [26, 27] - Family structures - Historical and contemporary social power relations [6] - Relevant changes over time (e.g. social changes or social movements ([63](#_ENREF_63)) |
| Socio-economic | This domain comprises the economic resources of a community and the access of a population to these resources [7, 28]. It also shows the relationship between an economy and its society. | Depending on the specific intervention, describing this domain may include:  **Refinement according to concept analysis:**   - Fiscal environment [14] - Market environment [17]   **Initial framework as based on the scoping review:**   - Social or socio-economic status [20], attributed to education, income, occupation marriage, or gender - Financial aspects (income, wealth, poverty) [5, 10] - Economic climate (e.g., reliance upon and stability of private, state, federal funding), [8, 14] - Education - Occupational aspects (employment status, working conditions) [5] - Living conditions (housing, neighbourhood characteristics) [29] - Relevant changes over time (e.g. inflation, recession, economic crisis) |
| Political | The political domain focuses on the distribution of power, assets and interests within a population, as well as the range of organisations involved, their interests and the formal and informal rules that govern interactions between them [30]. The domain also comprises the health care system and the securing of its accessibility. | Depending on the specific intervention, describing this domain may include:  **Refinement according to applications:**   - Access to health care system   **Refinement according to concept analysis:**   - Ideology [31] - Short-term thinking[31] - Influential people [31] - Payer, donor or funder policies [31, 32] - Political authority [17] - Health Care System [31-35] (e.g. governance and leadership, resources, service delivery, integration of patient’s needs and perspective)   **Initial framework as based on the scoping review:**   - Political system and civil society’s structure - political or social climate (e.g., liberal versus conservative) [8] - Players, interests, resources, objectives, formal and informal rules - Distribution of power - public policies (e.g., presence of state laws that criminalize HIV disclosure), [8, 34] - Political culture and socio-political climate [36] - State-society relations - Political situation including political stability and absence of violence, government effectiveness, voice and accountability, control of corruption, rule of law, regulatory quality, participation, accountability, transparency, efficiency, decency, and fairness[31, 32] - Economic management, economic policy and political framework of markets - Politics and gender - International integration [30] - Relevant changes over time (e.g. political reform, change of government) |
| Legal | The legal domain is concerned with the rules and regulations that have been established to protect a population‘s rights and societal interests [37]. | Depending on the specific intervention, describing this domain may include:  **Refinement according to applications:**   - Decision-making in care delivery - Sharing of information with indirectly affected stakeholders   **Refinement according to concept analysis:**   - Legislation [32, 34]   **Initial framework as based on the scoping review:**   - Norms, values and beliefs underlying legislation - Specific legislation (e.g. patient rights, data protection, malpractice liability) [34] [32] - Regulatory provisions concerning healthcare personnel and their rights and duties - Guidelines [28] - Relevant changes over time (e.g. introduction of new regulation or legislation) |
| Ethical | The ethical domain embraces reflections of morality, which encompasses beliefs, standards of conduct and principles that guide the behaviour of individuals and institutions [37]. Ethical issues at stake or in conflict, within systematic reviews/health technology assessments on a complex intervention, are addressed. | Depending on the specific intervention, describing this domain may include:  **Refinement according to applications:**   - **Autonomy**: Personal autonomy is, at minimum, self-rule that is free from both controlling interference by others and from limitations, such as inadequate understanding, that prevent meaningful choice. The autonomous individual acts freely in accordance with a self-chosen plan, analogous to the way an independent government manages its territories and sets its policies [38] - **Moral distress:** a phenomenon in which one knows the right action to take, but is constrained from taking it [39] - **Privacy**: privacy is a fundamental human right and need [40]. Privacy can be physical, social, psychological, and informational [41] - **Conflicting interests**: a set of circumstances that creates a risk that professional judgment or actions regarding a primary interest will be unduly influenced by a secondary interest.[42]   **Refinement according to concept analysis:**   - none   **Initial framework as based on the scoping review:**   - **Morality and beliefs:** Morality and beliefs influencing the behaviours of individuals in their private or professional capacity, and/or of institutions [11, 37] - **Ethical principles and code of conduct:** Standards of conduct and principles guiding the behaviours of individuals in their private or professional capacity, and/or of institutions [37] - Relevant changes over time (e.g. changes in standards of conduct within a healthcare organisation) |

**Table 2**: Domains of Implementation Dimension

| **Domain** | **Definition** | **Aspects** |
| --- | --- | --- |
| Implementation theory | An implementation theory attempts to explain the causal mechanisms of implementation [43] | - **Causal mechanisms of implementation** |
| Implementation process | The implementation process refers to the social processes, through which interventions are operationalized in an organization or community [44] | - **Exploration** [45, 46]: exploration of organizational needs, intervention-organizational fit as well as capacity and readiness assessment in a given setting [45, 46]. - **Decision to adopt** - **Planning and preparation**: a stage during which specific implementation strategies, the engagement of implementation agents as well as the implementation process itself are planned [47-49]. - **Initial implementation:** At this stage, staff should be educated and information disseminated; interventions can be pilot tested and consequently adapted [50][49-52] - **Full implementation [49, 51]:** processes and procedures supporting the intervention execution are in place, and the system, although never completely stable, has largely been recalibrated to accommodate and support the intervention [49] - **Evaluation and reflection** [45, 48, 52, 53]**:** The stage of evaluation and reflection aims to assess the process as well as the outcomes with reference to the intended goals [52] - **Sustainment** [46, 47, 51]**:** the continued use of the intervention in the organisation or community |
| Implementation strategies | Implementation strategies encompass all methods and means to ensure the adoption and sustainment of interventions [54, 55]. They comprise a set of activities that are chosen and tailored to fit a specific context [34, 56] or to create such a context [34] | - **Name of implementation strategy:** name or label of implementation strategy, preferably according to literature [54] - **Definition of implementation strategy:** Definition of implementation strategy and any discrete components operationally [54] - **Specification of implementation strategy** [54]**:**    - **Theory:** empirical, theoretical, or pragmatic justification for the choice of implementation strategies [54]   - **Actor:** who enacts the implementation strategy [54]   - **Action:** specific actions, steps, or processes that need to be enacted [54]   - **Action Target:** specification of targets according to conceptual models of implementation [54]   - **Temporality:** point of time at which implementation strategy is used [54]   - **Dose:** dosage of implementation strategy [54]   - **Implementation outcome affected**  [54] |
| Implementation agents | Implementation agents comprise all individuals and organisations engaged with (i) deciding to implement a given intervention (e.g. funders, administrators), (ii) implementing this intervention (e.g. providers, advocates, physicians, nurses) or (iii) being the target or otherwise affected by an intervention (e.g. patients and their families, consumers) [37] | - **Skills** [36, 57]: An ability or proficiency acquired through training and/or practice [57, 58]; (e.g. physical, cognitive and interpersonal skills) - **Knowledge** [57]: An awareness of the existence of something [57], Knowledge (including knowledge of condition /scientific rationale), Procedural knowledge, Knowledge of task environment [57] - **Social/professional role and identity** [57]: The behaviour considered appropriate for a particular kind of work or social position [57] - **Beliefs about capabilities and self-efficacy** [57]: Perceived behavioural control refers to people's perceptions of their ability to perform a given behaviour [59] - **Optimism**: The confidence that things will happen for the best, or that desired goals will be attained [57, 58] - **Beliefs about consequences** [57]: Acceptance of the truth, reality, or validity about outcomes of a behavior in a given situation [57] - **Reinforcement** [57]**:** Increasing the probability of a response by arranging a dependent relationship, or contingency, between the response and a given stimulus [57] - **Intentions** [57]**:** A conscious decision to perform a behavior or a resolve to act in a certain way [57] - **Emotion** [57]: A complex reaction pattern, involving experiential, behavioral, and physiological elements, by which the individual attempts to deal with a personally significant matter or event (e.g. Fear, Anxiety, Affect, Stress, Depression, Positive/negative affect, Burn‐out [57]) - **Goals** [36, 57]: Mental representation of outcomes or end states that an individual wants to achieve - **Memory, attention and decision processes** [57]: The ability to retain information, focus selectively on aspects of the environment, and choose between two or more alternatives [57] - **Behavioural regulation** [57]: Anything aimed at managing or changing objectively observed or measured actions (e.g. Self‐monitoring, Breaking habit, Action planning) [57] - **Attitude towards intervention**: The learned, relatively stable tendency to respond to people, concepts, and events in an evaluative way (78) - **Personality attributes** [28] (e.g. openness, curiosity) |
| Implementation outcomes | An implementation outcome is the result or implication of the implementation effort and forms part of good monitoring and evaluation practices. | - **Fidelity** [8, 52, 60]:  degree to which an intervention was implemented as it was prescribed in the original protocol or as it was intended by the program developers [61] - **Uptake** or adoption [62, 63]:  intention, initial decision, or action to try or employ an innovation or evidence-based practice [61] - **Acceptability** [63]: the perception among implementation stakeholders that a given treatment, service, practice, or innovation is agreeable, palatable, or satisfactory [61] - **Implementation cost** [8]: financial impact of an implementation effort [61] - **Penetration** [8]:  the integration of a practice within a service setting and its subsystems [61] - **Sustainability** [8, 28]:  the extent to which a newly implemented treatment is maintained or institutionalized within a service setting’s ongoing, stable operations [61] - **Dissemination to other contexts** [28] |

**Table 3**: Setting Dimension

| **Domain** | **Definition** | **Aspects** |
| --- | --- | --- |
| Setting | Setting refers to the specific physical location, in which the intervention is put into practice and interacts with context and implementation [55] | Depending on the specific intervention, describing this domain may include:  **Refinement according to applications:**   - Effect of location on affected stakeholder   **Refinement according to concept analysis:**   - physical characteristics [1] - work environment [2]   **Initial framework as based on the scoping review:**   - City, region, country (e.g. urban vs. rural, state, country, region) [3] [4] - Type of study site (e.g. primary care, hospital, home, school, occupational setting [3] - Number of study sites (e.g. number of primary care units, schoools) [3, 4] - Relevant changes over time (e.g. urbanisation) |
