# Supplementary material for: Making sense of complexity in context and implementation: the Context and Implementation of Complex Interventions (CICI) framework
Source: Implement Sci. 2017 Feb 15;12:21. doi: 10.1186/s13012-017-0552-5 (PMC5312531; doi:10.1186/s13012-017-0552-5)
Supplement: Additional file 5: — Data Extraction Tool for Qualitative Systematic Reviews. (DOC 55 kb) [file 13012_2017_552_MOESM5_ESM.doc]

# ***Additional File 5: Data Extraction Tool for Qualitative Systematic Reviews***

## ***Part A: Context***

- Which aspects of context interact with the implementation of the intervention?
- How do these aspects of context interact with the intervention?
- How do these aspects of context interact with implementation?

| **Domains** | | **Coding Levels** | |
| --- | --- | --- | --- |
|  |  | **Coding Level 1: Verbatim quotations (please indicate by *italics*) and participant** | **Coding Level 2: Interpretations of authors** |
| Geographical | - Geography (e.g. altitude, desert, forest, water) - Climate (e.g. temperature, rainfall) - Human land use (e.g. degree of urbanisation, agriculture, industry) - Infrastructure (e.g. water and sanitation, energy, transport) - Access to health care system - Geographical isolation - Relevant changes over time (e.g. infrastructure development, crop failures) |  |  |
| Epidemiological | - Demographics (life expectancy, gender, age, ethnicity, genetic factors) - Population density, fertility patterns, family size - Incidence/prevalence and severity of disease, morbidity and mortality - Spatial distribution of disease across geographical areas - Relevant changes over time (e.g. epidemics) |  |  |
| Socio-economic | - Social or socio-economic status attributed to education, income, occupation marriage, or gender - Financial aspects (income, wealth) - Occupational aspects (employment status, working conditions) - Living conditions (housing, neighbourhood characteristics) - Determinants of needs of people directly affected by disease/condition - Burden of disease - Determinants of needs of people indirectly affected by disease/condition - Fiscal environment - Market environment - Access to health care system - Relevant changes over time (e.g. inflation, recession, economic crisis) |  |  |
| Socio-cultural | - Language and means of communication - Symbols, heroes, rituals - Values (e.g. evil vs. good, dirty vs. clean, dangerous vs. safe, abnormal vs. normal) - Beliefs (e.g. superstition, fate or destiny) - Religiosity and spirituality - Knowledge and perceptions (e.g. with respect to significance of health issue, options for resolving health issue, multiple benefits and drawbacks of technology) - Lifestyle (population’s patterns in nutrition, smoking, substance abuse) - Discrimination - Social capital and resources available through social relationships, specifically social networks, norms of reciprocity, and trust - Social cohesion, including relational, material, and political dimensions, information exchange, networks of support, and informal social control - Historical and contemporary social power relations - Sociodemographic profiles - Psychosocial factors - Social and societal context - Structural social inequalities (e.g. Gender inequalities, caste system) - Community characteristics and level of coordination/involvement with community - Relevant changes over time (e.g. social changes or social movements) |  |  |
| Political | - Political system and civil society’s structure - Players, interests, resources, objectives, formal and informal rules - Distribution of power - Political culture and socio-political climate - State-society relations - Political situation including political stability and absence of violence, government effectiveness, voice and accountability, control of corruption, rule of law, regulatory quality, participation, accountability, transparency, efficiency, decency, and fairness - Economic management, economic policy and political framework of markets - Politics and gender - International integration - Ideology - Short-term thinking - Influential people - Payer, donor or funder policies - Political authority - Health Care System (e.g. governance and leadership, resources, service delivery, integration of patient’s needs and perspective) - Access to health care system - Relevant changes over time (e.g. political reform*, change of government) |  |  |
| Legal | - Norms, values and beliefs underlying legislation - Specific legislation (e.g. patient rights, data protection) - Regulatory provisions concerning healthcare personnel and their rights and duties - Guidelines - Decision-making in care delivery - Sharing of information with indirectly affected stakeholders - Legislation - Relevant changes over time (e.g. introduction of new regulation or legislation) |  |  |
| Ethical | - Refinement according to applications: - Autonomy - Moral distress - Privacy - Conflicting interests - Morality and beliefs - Ethical principles and code of conduct - Relevant changes over time |  |  |

## ***Part B: Implementation***

- Which aspects of [insert domain] were reported in the study?
- How does the [insert domain] interact with the setting and the context?
- How does the [insert domain] interact with the intervention?

| **Domains** | | **Coding Levels** | |
| --- | --- | --- | --- |
|  |  | **Coding Level 1: Verbatim quotations (please indicate by *italics*) and participant** | **Coding Level 2: Interpretations of authors** |
| Implementation theory | - Causal mechanisms of implementation |  |  |
| Implementation process | - Exploration - Planning and preparation - Initial implementation - Full implementation - Evaluation and reflection - Sustainment |  |  |
| Implementation strategies | - Name of implementation strategy - Definition of implementation strategy: - Specification of implementation strategy - Theory - Actor - Action - Action Target - Temporality - Dose - Implementation outcome affected |  |  |
| Implementation agents | - Skills - Knowledge - Social/professional role and identity - Beliefs about capabilities and self-efficacy - Optimism - Beliefs about consequences - Reinforcement - Intentions - Emotion - Goals - Memory, attention and decision processes - Behavioural regulation - Attitude towards intervention - Personality attributes |  |  |
| Implementation outcomes | - Fidelity - Adoption - Uptake - Acceptability - Implementation cost - Penetration - Sustainability - Dissemination to other contexts |  |  |

## ***Part C: Setting***

- Which aspects of setting were reported in the study?
- Which aspects of the setting interact with the intervention?
- How does the setting interact with the intervention?
- How does the setting interact with the context?
- How does the setting interact with the implementation?

| **Domains** | | **Coding Levels** | |
| --- | --- | --- | --- |
|  |  | **Coding Level 1: Verbatim quotations (please indicate by *italics*) and participant** | **Coding Level 2: Interpretations of authors** |
| Setting | - City, region, country (e.g. urban, rural) - Type of study site (e.g. primary care, hospital, home, school, occupational setting - Number of study sites - Physical characteristics - Work environment - Effect of location on affected stakeholder - Relevant changes over time (e.g. urbanisation) |  |  |
