# Supplementary material for: Making sense of complexity in context and implementation: the Context and Implementation of Complex Interventions (CICI) framework
Source: Implement Sci. 2017 Feb 15;12:21. doi: 10.1186/s13012-017-0552-5 (PMC5312531; doi:10.1186/s13012-017-0552-5)
Supplement: Additional file 6: — Expert Consultation Guide. (DOC 57 kb) [file 13012_2017_552_MOESM6_ESM.doc]

# ***Additional File 6***: Expert Consultation Guide

| **Item** | **General question (according to domains of CICI framework)** | **Concrete question** | **Demonstration of example** | **Follow-up question (implementation strategies** |
| --- | --- | --- | --- | --- |
| ***A*** | **Interviewer (I): “Context** reflects a set of characteristics and circumstances that consist of active and unique factors, within which the implementation is embedded” | | | |
| A.1 | Which **contextual** factors may influence the implementation of *the intervention* in your country?  *Notes:*  *The CICI framework contains the following context domains:*  *geographical, epidemiological, socio-cultural, socio-economic, ethical, legal, political* | Which *[insert domain]* factors may affect the implementation of *the intervention* in your country? | Our research has shown that [insert example of domain] affects the implementation.   - Does this also apply in your country? - Do you consider this an advantage/disadvantage to the implementation? | Optional:  Which implementation strategies could be employed in order to overcome these *[insert domain]* barriers/strengthen these *[insert domain]* facilitators? |
| A.2 |  | Can you think of any other *[insert domain]* factors that may hinder or facilitate the implementation of *the intervention*? |  | Optional:  Which implementation strategies could be employed in order to overcome these *[insert domain]* barriers/strengthen these *[insert domain]* facilitators? |
| A.3 |  | How do *[insert domain]* aspects of context interact with the intervention? | Our research has shown that [insert example of domain] interacts with the intervention.   - Does this also apply in your country? - Do you consider this an advantage/disadvantage to the implementation? | Optional:  Which implementation strategies could be employed in order to overcome these *[insert domain]* barriers/strengthen these *[insert domain]* facilitators? |
| A.4 |  | How do *[insert domain]* aspects of context interact with implementation? |  |  |
| B | “The **implementation process** is an active, multistage, iterative and dynamic process that does not usually occur in a linear fashion. “ | | | |
| B.1 | Which steps of the **implementation** **process** are passed through and how may they influence the intervention in your country? | Which stages of the implementation process are passed through during implementation? | Our research has shown that [insert steps] are passed through during implementation.   - Does this also apply in your country? - Do you consider this an advantage/disadvantage to the implementation? |  |
| B.2 |  | How does the implementation process interact with the setting and the context? | Our research has shown that [insert steps of implementation process] interacts with context and setting.   - Does this also apply in your country? - Do you consider this an advantage/disadvantage to the implementation? |  |
| B.3 |  | How does the implementation process interact with the intervention? | Our research has shown that [insert steps of implementation process] interacts with the intervention.   - Does this also apply in your country? - Do you consider this an advantage/disadvantage to the implementation? |  |
| C | “Whenever an intervention is implemented, specific **implementation strategies** are employed in order to achieve a successful intervention. Implementation strategies encompass all methods and means to ensure the adoption and sustainment of interventions.” | | | |
| C.1 | Which and how may i**mplementation strategies** influence the intervention in your country? | Which implementation strategies are employed during implementation? | Our research has shown that [insert implementation strategy/ies] have/has been employed during implementation.   - Does this also apply in your country? - Do you consider this an advantage/disadvantage to the implementation? |  |
| C.2 |  | How do these implementation strategies interact with the setting and the context? | Our research has shown that [insert implementation strategies] interact(s) [insert way of interaction] with the setting and the context.   - Does this also apply in your country? - Do you consider this an advantage/disadvantage to the implementation? |  |
| C.3 |  | How do these implementation strategies interact with the intervention? | Our research has shown that [insert implementation strategies] interact(s) [insert way of interaction]with the intervention.   - Does this also apply in your country? - Do you consider this an advantage/disadvantage to the implementation? |  |
| D | “An intervention is usually implemented by individuals and organisations. These are engaged with (i) deciding to implement a given intervention (e.g. funders, administrators), (ii) implementing this intervention (e.g. providers, advocates, physicians, nurses) or (iii) are targeted or otherwise affected by an intervention (e.g. patients and their families, consumers).” | | | |
| D.1 | Which and how may **implementation agents** influence the intervention in your country? | Which implementation agents are involved in the implementation effort? | Our research has shown that [insert implementation agents(s)] is/are involved in the implementation effort.   - Does this also apply in your country? - Do you consider this an advantage/disadvantage to the implementation? |  |
| D.2 |  | How do these implementation agents interact with the setting and the context? | Our research has shown that [insert implementation agents(s)] interacts [insert way of interaction] with the implementation effort.   - Does this also apply in your country? - Do you consider this an advantage/disadvantage to the implementation? |  |
| D.3 |  | How do these implementation agents interact with the intervention? | Our research has shown that [insert implementation agents(s)] interacts [insert way of interaction] with the intervention.   - Does this also apply in your country? - Do you consider this an advantage/disadvantage to the implementation? |  |
| **E** | **Setting** refers to the specific physical location, in which the intervention is put into practice and interacts with context and implementation. | | | |
| E.1 | Which and how many aspects of the **setting** in which the intervention is implemented influence your intervention? | Which aspects of the setting interact with the intervention? | Our research has shown that [insert aspect of setting] interacts with the intervention.   - Does this also apply in your country? - Do you consider this an advantage/disadvantage to the implementation? |  |
| E.2 |  | How does the setting interact with the intervention? | Our research has shown that [insert aspect of setting] interacts [insert way of interaction] with the intervention.   - Does this also apply in your country? - Do you consider this an advantage/disadvantage to the implementation? |  |
| E.3 |  | How does the setting interact with the context? | Our research has shown that [insert aspect of setting] interacts [insert way of interaction] with the context.   - Does this also apply in your country? - Do you consider this an advantage/disadvantage to the implementation? |  |
| E.4 |  | How does the setting interact with the implementation? | Our research has shown that [insert aspect of setting] interacts [insert way of interaction] with the implementation.   - Does this also apply in your country? - Do you consider this an advantage/disadvantage to the implementation? |  |
