# Supplementary material for: Making sense of complexity in context and implementation: the Context and Implementation of Complex Interventions (CICI) framework
Source: Implement Sci. 2017 Feb 15;12:21. doi: 10.1186/s13012-017-0552-5 (PMC5312531; doi:10.1186/s13012-017-0552-5)
Supplement: Additional file 7: — Worked example of three frameworks (CICI, CFIR, PARIHS). (DOCX 143 kb) [file 13012_2017_552_MOESM7_ESM.docx]

# *Additional File 7: Worked example of three frameworks (CICI, CFIR, PARIHS)*

In order to demonstrate the added value of the context and implementation of complex interventions (CICI) framework when compared to other commonly used frameworks, we are providing a worked example: We applied the CICI framework, the Consolidated Framework for advancing Implementation Research (CFIR) [1] and the Promoting Action on Research Implementation in Health Services (PARIHS) framework [2] to the Air Pollution Act on the Marketing, Sale and Distribution of Coal, as enacted in the city of Dublin in 1990 [3]. We chose this intervention because it is evaluated in one of the studies [4] that we included in an ongoing Cochrane systematic review of interventions to reduce particulate matter air pollution and their effect on health [5].

The original Act was passed in 1987 (Air Pollution Act, and implemented in the city of Dublin from September 1, 1990 [4]. In the following years, the original act was extended to other cities and several amendments were made (e.g. increase of penalties, limits on sulphur content in bituminous coal). Since 2012, not only the sale, marketing and distribution, but also the burning of specified fuels has been prohibited; at the same time, cleaner alternatives, such as natural gas were promoted. The last amendments were made in 2015 (extension to additional areas, inclusion of public houses in covered areas). Bans were introduced in urban settings (>15,000 inhabitants), and currently, 27 cities are covered by the ban [6]. The logic model in Figure 1 shows the relevant participants, the intervention (according to theory, design and delivery) as well as expected environmental, health and non-health outcomes.


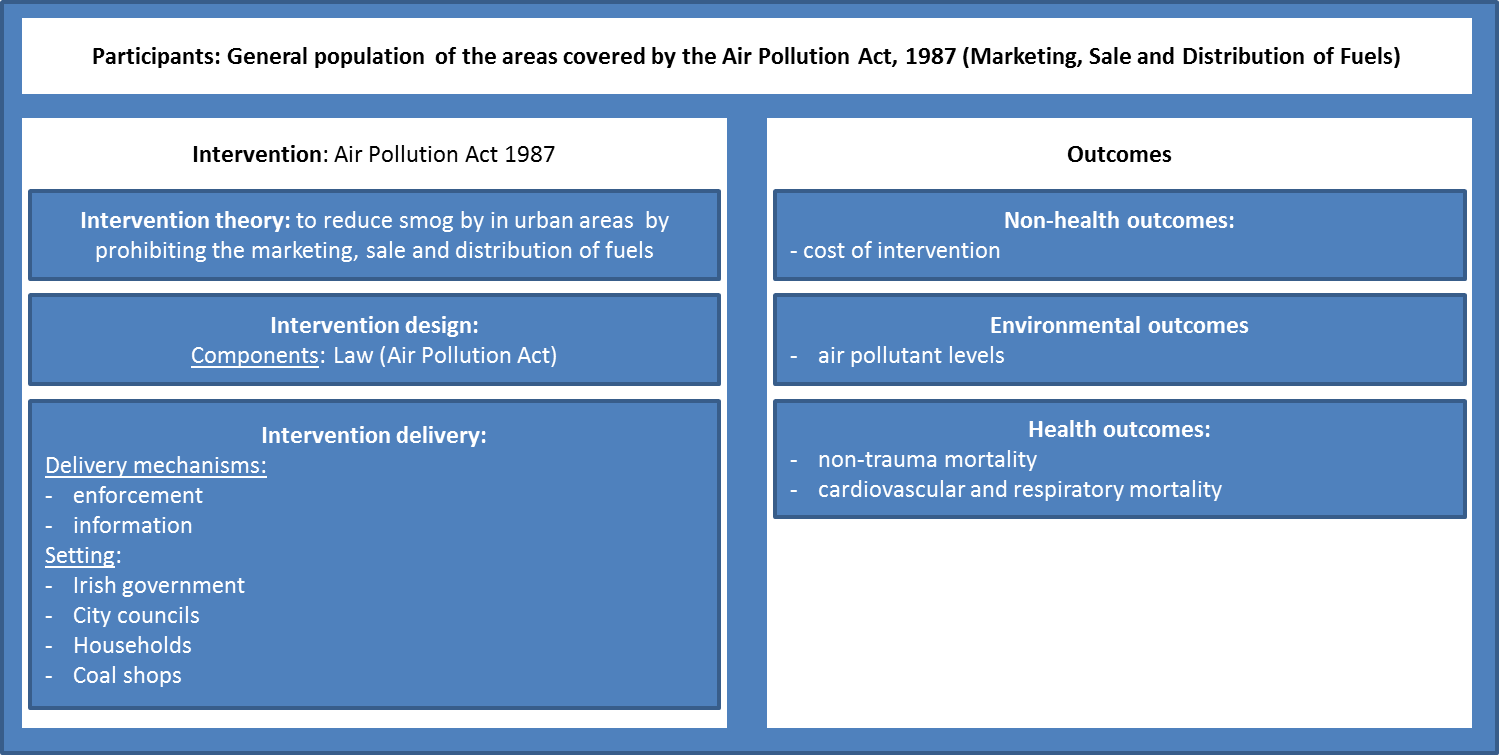


Figure 1: Logic model of Air Pollution Act on the Marketing, Sale and Distribution of Coal

As a starting point for populating the three frameworks, we used the study included in the above mentioned Cochrane review, a detailed report by the Health Effects Institute (Dockery et al (2013). In addition to using the information therein, we conducted specific searches of the grey literature and identified relevant information in newspaper articles, government documents, city council reports as well as lobbyism reports and summarized the retrieved information under the relevant domains of the three frameworks.

## Application of CICI

| Context | | | | Interactions |
| --- | --- | --- | --- | --- |
|  | Socio-economic context | This domain comprises the economic resources of a community and the access of a population to these resources [7, 8]. It also shows the relationship between an economy and its society. | Macro (National and International level)   - **Economic changes:** Ireland has undergone major changes in industry, switching from traditional industries to service industries (knowledge economy). The interventions in Ireland were happening when the Irish economy was the fastest growing in Europe - **Wealth:** In terms of gross domestic product, Ireland ranks among the top countries in the OECD - **Import of coal:** the industry importing coal might be affected by the introduction of the coal ban [9] - **Solid Fuel Trade:** Decline of solid fuel sales after introduction of ban; the government expected that jobs would not be affected by ban since traders could switch to selling non-banned solid fuels. [9]   Meso (City level)   - **Industrial hub:** Historically, Dublin was the largest industrial center, although service industries currently dominate the economy. [4] - **Import of coal:** Dublin was the main center for the importation of coal, and the local ban in Dublin might have affected availability in other cities; However, following the ban, the solid fuel market and industry has suffered in the Dublin area.[9]   Micro (Household level, solid fuel traders)   - **Loss of jobs:** potential loss of jobs in solid fuel vendors [9] - **Reliance on polluting fuels:** Families of lower socio-economic status have heating that relies on coal due to the cost of the fuel and cost of adaptation to another heating system (e.g. gas) - **Price of alternative fuels:** The price of smokeless coal was a barrier for some to ensuring a smoke-free home[10] | Political context  Legal context  Socio-cultural context  Ethical context  Implementation agents  Implementation strategy  Implementation process  Implementation outcomes |
|  | Socio-cultural context | This domain comprises explicit and implicit behaviour patterns, including their embodiment in symbols and artefacts; the essential core of culture consists of historically derived and selected ideas and values that are shared among members of a group [11]. It not only refers to the conditions in which people are born, grow, live, work and age but also embraces the social roles a human being takes on as a family member, community member or citizen and the relationships inherent to these roles. Constructs such as knowledge, beliefs, conceptions, customs, institutions and any other capabilities and habits acquired by a group are covered by this domain [12]. | Macro (National level):   - **Coal as traditional fuel:** Coal has been used for heating for many years [13]   Meso (City level):   - **Coal as traditional fuel:** Coal as “part of Dublin’s heritage” [14]   Micro (Household level):   - **Preference for open fire**: “People in Ireland like the open fire” [15] | Socio-economic context |
|  | Ethical context | The ethical domain embraces reflections of morality, which encompasses beliefs, standards of conduct and principles that guide the behaviour of individuals and institutions [16]. Ethical issues at stake or in conflict, within systematic reviews/health technology assessments of a complex intervention, are addressed. | Meso level:   - **Inequity:** Neighbourhoods with low socio-economic status are disproportionally affected by smog because people living in these communities buy cheaper coal - **Loss of jobs:** potential loss of jobs in solid fuel traders [9] | Socio-economic context  Political context  Implementation process  Implementation strategy |
|  | Legal context | The legal domain is concerned with the rules and regulations that have been established to protect a population‘s rights and societal interests [16]. | Macro level:   - **European Commission Directive:** As part of the CAFE Directive (2008/50/EC), Ireland must reduce its average PM2.5 background concentration by 10% by 2018 - **Incentives**: Introduction of carbon tax on solid fuels [17] | Political  Implementation strategies  Implementation agents |
|  | Political context | The political domain focuses on the distribution of power, assets and interests within a population, as well as the range of organisations involved, their interests and the formal and informal rules that govern interactions between them [18]. The domain also comprises the health care system and the securing of its accessibility. | Macro level:   - **Political Division:** The island of Ireland is historically divided into 32 counties. Of these, 26 are in the Republic of Ireland and 6 are in Northern Ireland.[4] - **Oil Crisis:** In the period of spiralling oil prices in the 1970s, the Republic of Ireland provided Government grants to encourage its citizens to switch to coal fires - **Smog attenion:** Extremely high levels of smog in Dublin alerted public health and environment professionals [10]; by the late 1980s, the smog issue was firmly on the public agenda [10] - **Environmental activism:** The situation prompted environmentalists to distribute hundreds of free surgical masks, as opposition politicians warned that people could die unless the Government effectively combated the persistent pall. - **Lobbyism:** Coal Information Service – the lobbying arm of the coal industry – maintained coal was “part of Dublin’s heritage” [14]; however, Ireland’s ability to substitute gas for coal was made easier because it benefited from gas supply security and from the lack of a large domestic coal industry that could have lobbied against the ban [19] - **Clean Heating Initiatives:** Sustainable Energy Authority of Ireland (SEAI) incentivise the improvement of the building stock through various schemes such as the “Better Energy Homes Scheme”, “The Warmer Homes Scheme”, “The Better Energy Communities Scheme” and others | Legal  Socio-economic  Geographical  Epidemiological |
|  | Epidemiological context | This domain refers to the distribution of disease/conditions, the attributable burden of disease as well as determinants of needs in human populations [20]. Therefore, it also includes demographics [21, 22]. | Meso context:   - **Population:** Dublin County Borough, that is, the city of Dublin, had a population of 478,389 in 1991 and of 525,383 in 2011 [23]; Dublin had a population density of 3,498 people/km2 in 2011 [9] - **Health outcomes:** Respiratory mortality decreased significantly, by 17%, after the 1990 ban (confirming the earlier study) and, to a lesser extent, after the 1995 and 1998 bans. However, unlike the earlier study, the current study did not find a reduction in total or cardiovascular mortality after either the 1990 ban or the later bans [4] - **Air pollution:** Dockery et al (2013) found decreases in black smoke concentrations, particularly during the heating season, after each ban. [4] | Geographical  Political  Implementation process |
|  | Geographical context | The geographical characteristics refer to the broader physical environment, landscapes and resources, both natural and transformed by humans, available at a given location. As such it also comprises the infrastructure at a given location, which could result in geographical isolation. | Macro   - **Air Pollution:** During the 1980s, the Republic of Ireland experienced repeated severe pollution episodes [4]; The smog choked the city mercilessly for about a week in November, with amounts nearly seven times above pollution levels considered worrisome by the World Health Organization[10] - **Urban areas**: Bans were introduced in urban settings only [6]; many of these cities are located at the coast of Ireland - **Temperature inversion:** Cities are prone to temperature inversion (leading to smog) due to their geographical location [15]   Meso:   - **Industrial hub:** Dublin is the hub of the nation’s roadway system, and thus traffic, including both light- and heavy-duty vehicles, which constitute a major source of pollution | Political context  Socio-economic context  Implementation Strategy  Implementation Process  Implementation Agents |
| Implementation | | | |  |
|  | Implementation theory | An implementation theory attempts to explain the causal mechanisms of implementation [24] | - **Multiple levels:** Policy and regulatory measures to reduce particulate matter in urban areas are enforced on an international, national, regional and micro (household, vendor) level. - **Enforcement and information:** The underlying theory is that the Air Pollution Act, a legal intervention, is implemented through enforcement and information, which influences both industry (in particular coal vendors) and households in terms of selling and using polluting fuels, respectively. | Implementation agents  Implementation strategy  Implementation process  Political context  Legal conext |
|  | Implementation process | social processes, through which interventions are operationalized in an organization or community [25] | Macro and Meso:   - **Exploration**: In 1956, the city of London also made use of a Clean Air Act in response to the Great Smog of 1952 (introduction of smoke control areas in which only smokefree fuels could be burned); During the smog crisis in Dublin, several programmes were in place (e.g. to equip local authority houses and flats with smokeless heating systems; information campaign to encourage the use of smokeless fuels and heating systems was being prepared (March 1989), introduction of a smog alert system). - **Decision to adopt**: an act to provide for the control of air pollution and other matters connected with air pollution. [10th june, 1987] - **Planning and preparation**: Planning and preparation was done between 1987 and 1990 when the ban was introduced in Dublin - **Initial implementation**: The first ban was introduced in Dublin in 1990 following severe episodes of winter smog caused by the use of fuels for home-heating. - **Full implementation**: In 1995, 1998, 2000, 2003 and 2011, 2015 the ban was extended to other urban areas - **Evaluation and reflection**: Research indicated that the ban in Dublin was effective immediately [26]; In 2012, review and public consultation on the ban of smoky coal in parts of the country in order to “review and modernise” the ban [27] - **Sustainment**: there are plans to extend the Act to the entire Republic of Ireland | Epidemiological context |
|  | Implementation strategy | Implementation strategies encompass all methods and means to ensure the adoption and sustainment of interventions [28, 29]. They comprise a set of activities that are chosen and tailored to fit a specific context [30, 31] or to create such a context [30] | Macro:   - **Grants:** Grants for heating adaptations are provided to individual households [9] - **Tacation**: Introduction of carbon tax on solid fuels [17]   Meso:   - **Enforcement**: Inspection of premises and vehicles used for the sale and distribution of solid fuel as well as collect samples by authorised local authority staff [3]; Environmental health officer (EHO) in the Air Quality and Noise Control Unit have maintained a presence on the streets of Dublin enforcing the legislation since it was first introduced. They carry out inspections of fuel yards, fuel vehicles, shops and garage forecourts [32] - **Prosecution:**   - **Fines:** A local authority may bring a prosecution under the Air Pollution Act for breaches of the Regulations. [33]; The maximum fine amounts for breaches of the Regulations is 5,000 euro on summary conviction. [33]   - **Fixed payment notices:** Fixed payment notices (or ‘on the spot fines’) have been introduced for alleged offences relating to the marketing, sale and distribution of prohibited fuels in specified areas. Persons found to be marketing, selling or distributing prohibited fuels in breach of the Regulations are now liable for a fixed payment notice of between 250 euro and 1,000 euro depending on the offence.[6] - **Local planning:**    - **Air Quality Management Plan:** Local authorities are required to make Air Quality Management Plans, containing objectives as seem to the local authority (or authorities) concerned to be reasonable and necessary for the prevention or limitation of air pollution or the preservation or improvement of air quality in their area [33]   - **Transmission of Air Quality Plan:** A local authority, having made an air quality management plan, must transmit a copy of the plan to the Minister of Environment Heritage and Local Government and to such other persons as prescribed in the Act. [33]   Micro:   - **Grants:** Grants for heating adaptations can be applied for by households [9] | Political context  Legal context |
|  | Implementation agents | Implementation agents comprise all individuals and organisations engaged with (i) deciding to implement a given intervention (e.g. funders, administrators), (ii) implementing this intervention (e.g. providers, advocates, physicians, nurses) or (iii) being the target or otherwise affected by an intervention (e.g. patients and their families, consumers) [37] | Macro:   - **Alerting professionals:** In 1988, the Minister for Health, O’Hanlon, alerted professionals to the situation [10] - **Collaboration between Ministers:** In 1989, Deputy Quinn spoke of his frustration about the situation; he questioned who was benefitting from the sale of the coal, and accused the Minister of State Harney, as being “totally negligent in the execution of her duties”, due to political and personal differences with the Minister for the Environment.[10] - **Collaboration between Ministries:** Minister of Health O’Hanlon said it was the Minister for the Environment who “has responsibility for statutory controls to protect the public and the environment from the effects of air pollution”, and that he himself had taken up the matter with the Minister of Environment, Harney. [10] - **Setting up a team:** A committee of ministers had been set up to focus on smog and its effect on public health. [10] - **Personal leadership**: Minister Flynn assured that a number of measures were in place, such as a programme to equip local authority houses and flats with smokeless heating systems. He also noted that he introduced a smog alert system. [10] “Smog has been around a long time and I am the first person who tried to do something about it,” - **Collaboration with industry:** The price of smokeless coal was a barrier for some to ensuring a smoke-free home, and Minister Flynn met with members of the fuel industry that led to a drop in price of such coal, and an increased availability and promotion of smokeless fuels [10] - **Collaboration with research entities:** financial contributions towards research, surveys or investigations connected with air pollution [3] - **Cooporation between Northern Ireland and the Republic of Ireland:** The study that showed the effectiveness of the ban is a joint piece of research between Northern Ireland and the Republic; (“North-South cooperation in this area provides an opportunity to further improve air quality for the citizens of this island both North and South,” [17] - **Supporting organisations:** Irish Environmental Protection Agency: oversees the enforcement of regulations with new legislation requiring the registration of coal baggers and suppliers [3]; Network for Ireland's Environmental Compliance and Enforcement(NIECE) - **Media**: Television and radio campaign (''Stop smog. Switch to smokeless.'') to encourage the use of smokeless fuels and heating systems was being prepared (March 1989) [10]   Meso:   - **Local authorities:**   - enforce regulations in covered areas by and appointing authorised persons to inspect premises and vehicles [33]   - make financial contributions towards research, surveys or investigations connected with air pollution (section 18 of the Air Pollution Act, 1987)   - Declare certain areas to be special control areas (section 39 of the Air Pollution Act, 1987)   - Make air quality management plans (section 46 of the Air Pollution Act, 1987)   Micro:   - **Individuals:**   - Individuals adapt to the regulation by changing their heating system   - Individuals find ways to buy coal outside of the areas covered by the Act   - Smog was so intense that it blocked sunlight for as long as two weeks [33] | Political context  Legal context  Socio-economic context  Socio-cultural context  Geographical context |
|  | Implementation outcomes | An implementation outcome is the result or implication of the implementation effort and forms part of good monitoring and evaluation practices. | - **Fidelity**: Anecdotal evidence suggests some residents were purchasing bituminous coal, available legally in local shops just outside the current ban area, to use within the coal ban area. [34] - **Acceptability**:   - For the public, the price of alternative fuels for heating, and the levels of assistance for making such a transition will largely determine acceptability [9]   - Political acceptance will largely be determined by the economic consequences of a ban, the availability of alternative fuels for consumers and the reliance of the residential sector on the fuel product to be banned [9] - **Diffusion** to other contexts: Due to the effectiveness of the coal ban, the ban was extended to other cities in Ireland. There are also to plans to extend the ban to the entire Republic of Ireland. [35] The intervention is now considered a good example for urban areas where heating with coal is standard practice[9] - **Sustainability**: an adapted version of the original ban from 1987 is still sustained in Dublin [4] | Epidemiological context  Socio-cultural context  Socio-economic context  Political context  Implementation agents  Implementation process |
| Setting | | | |  |
|  | Setting | The setting encompasses the immediate physical and organisational environment, in which an intervention is delivered. It also comprises the effect the location has on affected stakeholder, i.e. by taking on a specific role. | Macro:   - The Republic of Ireland with the Irish government passing the Air Pollution Act   Meso:   - The urban areas that are covered by the regulation, with the city council enforcing the regulation, providing information to vendors and households and reporting to the national government   Micro:   - The household that reacts to the regulation |  |

## Application of CFIR

| CFIR*  * In this case, we consider the respective local authorities that is intended to enforce the regulation as the organization of interest. | | | | | |
| --- | --- | --- | --- | --- | --- |
| Outer setting | | | |  |  |
|  | Patient needs & resources | | | The extent to which patient needs, as well as barriers and facilitators to meet those needs, are accurately known and prioritized by the organization. | - **Smog:** smog was so intense that it blocked sunlight for as long as two weeks [33] |
|  | Cosmopolitanism | | | The degree to which an organization is networked with other external organizations. | - **Network with research entities:** make financial contributions towards research, surveys or investigations connected with air pollution (section 18 of the Air Pollution Act, 1987) - **Network with EPA:** Make air quality management plans (section 46 of the Air Pollution Act, 1987) |
|  | Peer pressure | | | Mimetic or competitive pressure to implement an intervention; typically because most or other key peer or competing organizations have already implemented or are in a bid for a competitive edge | *Other cities might feel pressured by the effectiveness and success of the Dublin coal ban and consequently adapt this intervention* |
|  | External policy & incentives | | | A broad construct that includes external strategies to spread interventions, including policy and regulations (governmental or other central entity), external mandates, recommendations and guidelines, pay-for-performance, collaboratives, and public or benchmark reporting. | - **Policies:** External policies comprise all preceding policies and regulations that have been in place before the 2012 regulation has been installed; local authorities are required by the Air Pollution Act 1987 to make plans for preservation or the improvement of air quality in their functional area [33] - **Clean Heating Initiatives:** Sustainable Energy Authority of Ireland (SEAI) incentivise the improvement of the building stock through various schemes such as the “Better Energy Homes Scheme”, “The Warmer Homes Scheme”, “The Better Energy Communities Scheme” and others - **European Commission Directive:** As part of the CAFE Directive (2008/50/EC), Ireland must reduce its average PM2.5 background concentration by 10% by 2018 - **Taxation**: Introduction of carbon tax on solid fuels [17] - **Fixed payment notices:** Fixed payment notices (or ‘on the spot fines’) have been introduced for alleged offences relating to the marketing, sale and distribution of prohibited fuels in specified areas. Persons found to be marketing, selling or distributing prohibited fuels in breach of the Regulations are now liable for a fixed payment notice of between 250 euro and 1,000 euro depending on the offence.[6] |
| Inner setting | | | |  |  |
|  | Structural Characteristics | | | The social architecture, age, maturity, and size of an organization. | No information found |
|  | Networks & Communications | | | The nature and quality of webs of social networks and the nature and quality of formal and informal communications within an organization. | No information found |
|  | Culture | | | Norms, values, and basic assumptions of a given organization. | No information found |
|  | Implementation Climate | | | The absorptive capacity for change, shared receptivity of involved individuals to an intervention, and the extent to which use of that intervention will be rewarded, supported, and expected within their organization. | No information found |
|  |  | Tension for Change | | The degree to which stakeholders perceive the current situation as intolerable or needing change. | No information found |
|  |  | Compatibility | | The degree of tangible fit between meaning and values attached to the intervention by involved individuals, how those align with individuals’ own norms, values, and perceived risks and needs, and how the intervention fits with existing workflows and systems. | No information found |
|  |  | Relative Priority | | Individuals’ shared perception of the importance of the implementation within the organization. | No information found |
|  |  | Organizational Incentives & Rewards | | Extrinsic incentives such as goal-sharing awards, performance reviews, promotions, and raises in salary, and less tangible incentives such as increased stature or respect. | No information found |
|  |  | Goals & Feedback | | The degree to which goals are clearly communicated, acted upon, and fed back to staff, and alignment of that feedback with goals. | - **Air Quality Management Plan:** Local authorities are required to make Air Quality Management Plans, containing objectives as seem to the local authority (or authorities) concerned to be reasonable and necessary for the prevention or limitation of air pollution or the preservation or improvement of air quality in their area [33] - **Transmission of Air Quality Plan:** A local authority, having made an air quality management plan, must transmit a copy of the plan to the Minister of Environment Heritage and Local Government and to such other persons as prescribed in the Act. [33] |
|  |  | Learning Climate | | A climate in which: a) leaders express their own fallibility and need for team members’ assistance and input; b) team members feel that they are essential, valued, and knowledgeable partners in the change process; c) individuals feel psychologically safe to try new methods; and d) there is sufficient time and space for reflective thinking and evaluation. | No information found |
|  | Readiness for implementation | | | Tangible and immediate indicators of organizational commitment to its decision to implement an intervention. | - **Commitment**: City councils are legally obliged to respond as executive at meso level |
|  |  | | Leadership Engagement | Commitment, involvement, and accountability of leaders and managers with the implementation. | No information found |
|  |  | | Available Resources | The level of resources dedicated for implementation and on-going operations, including money, training, education, physical space, and time. | No information found |
|  |  | | Access to Knowledge & Information | Ease of access to digestible information and knowledge about the intervention and how to incorporate it into work tasks. | No information found |
| Characteristics of individuals | | | |  | - **Environmental health officer (EHO):** EHOs in the Air Quality and Noise Control Unit have maintained a presence on the streets of Dublin enforcing the legislation since it was first introduced. They carry out inspections of fuel yards, fuel vehicles, shops and garage forecourts [32] |
|  | Knowledge & Beliefs about the Intervention | | | Individuals’ attitudes toward and value placed on the intervention as well as familiarity with facts, truths, and principles related to the intervention. | No information found |
|  | Self-efficacy | | | Individual belief in their own capabilities to execute courses of action to achieve implementation goals. | No information found |
|  | Individual Stage of Change | | | Characterization of the phase an individual is in, as he or she progresses toward skilled, enthusiastic, and sustained use of the intervention. | No information found |
|  | Individual Identification with Organization | | | A broad construct related to how individuals perceive the organization, and their relationship and degree of commitment with that organization. | No information found |
|  | Other Personal Attributes | | | A broad construct to include other personal traits such as tolerance of ambiguity, intellectual ability, motivation, values, competence, capacity, and learning style. | No information found |
| Process | | | |  |  |
|  | Planning | | | The degree to which a scheme or method of behavior and tasks for implementing an intervention are developed in advance, and the quality of those schemes or methods. | - **Planning and preparation:** Planning and preparation was done between 1987 and 1990 when the ban was introduced in Dublin |
|  | Engaging | | | Attracting and involving appropriate individuals in the implementation and use of the intervention through a combined strategy of social marketing, education, role modeling, training, and other similar activities. | No information found |
|  |  | Opinion Leaders | | Individuals in an organization who have formal or informal influence on the attitudes and beliefs of their colleagues with respect to implementing the intervention. | No information found |
|  |  | Formally Appointed Internal Implementation Leaders | | Individuals from within the organization who have been formally appointed with responsibility for implementing an intervention as coordinator, project manager, team leader, or other similar role. | - Local authorities were required to appoint authorised persons to enforce the Act on a local level.[3] |
|  |  | Champions | | Individuals who dedicate themselves to supporting, marketing, and ‘driving through’ an implementation, overcoming indifference or resistance that the intervention may provoke in an organization. | No information found |
|  |  | External Change Agents | | Individuals who are affiliated with an outside entity who formally influence or facilitate intervention decisions in a desirable direction. | - **Collaboration with research entities:** financial contributions towards research, surveys or investigations connected with air pollution [3] - **Cooporation between Northern Ireland and the Republic of Ireland:** The study that showed the effectiveness of the ban is a joint piece of research between Northern Ireland and the Republic; (“North-South cooperation in this area provides an opportunity to further improve air quality for the citizens of this island both North and South,” [17] - **Supporting organisations:** Irish Environmental Protection Agency: oversees the enforcement of regulations with new legislation requiring the registration of coal baggers and suppliers [3]; Network for Ireland's Environmental Compliance and Enforcement(NIECE) - **Media**: Television and radio campaign (''Stop smog. Switch to smokeless.'') to encourage the use of smokeless fuels and heating systems was being prepared (March 1989) [10] |
|  | Executing | | | Carrying out or accomplishing the implementation according to plan. | - **Execution:** The ban became effective Dublin in 1990 |
|  | Reflecting & Evaluating | | | Quantitative and qualitative feedback about the progress and quality of implementation accompanied with regular personal and team debriefing about progress and experience. | - **Evaluation and reflection**: Research indicated that the ban in Dublin was effective immediately [26]; In 2012, review and public consultation on the ban of smoky coal in parts of the country in order to “review and modernise” the ban [27] |

## Application of PARiHS

| E: Evidence and evidence-based practice (EBP) Characteristics | | |  |
| --- | --- | --- | --- |
|  | Research and published guidelines |  | Research indicated that the ban in Dublin was effective immediately [26]; In 2012, review and public consultation on the ban of smoky coal in parts of the country in order to “review and modernise” the ban [27] |
|  | Clinical experiences and perceptions |  | Previous experiences with a coal ban in response to smog were made in London in the 1950s [10] |
|  | Patient experiences, needs, and preferences |  | Inhabitants of Dublin have been complaining about heavy smog (''Whenever we have a bad weekend of smog, the doctors' offices are packed') and environmentalists distributed hundreds of free surgical mask to protect citizens from smog [15] |
|  | Local practice information |  | Preference for coal as heating fuel and open fire “People in Ireland like the open fire” [13, 15] |
|  | Characteristics of the targeted EBP | |  |
|  |  | Relative advantage | No information found |
|  |  | Observability | No information found |
|  |  | Compatibility | No information found |
|  |  | Complexity | No information found |
|  |  | Trialability | No information found |
|  |  | Design quality and packaging | No information found |
|  |  | Costs | Mcloughlin (2001) undertook a cost benefit analysis of the coal ban and concluded that households that switched to oil or gas as opposed to an alternative solid fuel were better off over a 20-year time frame [9] |
| Contextual Readiness for Targeted EBP Implementation | | | |
|  | Leadership support |  |  |
|  |  | Clarify roles | No information found |
|  |  | Marketing | No information found |
|  |  | Communicating | No information found |
|  |  | Enabling effective teamwork | No information found |
|  |  | Enhancing collaboration | No information found |
|  |  | Providing general support | No information found |
|  |  | Ensuring accountability | No information found |
|  | Culture |  | No information found |
|  | Evaluation capabilities |  | - **Air Quality Management Plan:** Local authorities are required to make Air Quality Management Plans, containing objectives as seem to the local authority (or authorities) concerned to be reasonable and necessary for the prevention or limitation of air pollution or the preservation or improvement of air quality in their area [33] - **Transmission of Air Quality Plan:** A local authority, having made an air quality management plan, must transmit a copy of the plan to the Minister of Environment Heritage and Local Government and to such other persons as prescribed in the Act. [33] |
|  | Receptivity to the targeted innovation/  change |  | No information found |
| Facilitation | | | |
|  | Role of Facilitator |  | No information found |
|  | Purpose, external and/or internal role |  | No information found |
|  | Expectations and activities |  | No information found |
|  | Skills and attributes of facilitator |  | No information found |
| SI: Successful Implementation | | | |
|  | Implementation plan and its realization |  | - **Fidelity**: Anecdotal evidence suggests some residents were purchasing bituminous coal, available legally in local shops just outside the current ban area, to use within the coal ban area. [34] |
|  | EBP innovation uptake: uptake of clinical interventions and/or delivery system interventions |  | - **Acceptability**:   - For the public, the price of alternative fuels for heating, and the levels of assistance for making such a transition will largely determine acceptability [9]   - Political acceptance will largely be determined by the economic consequences of a ban, the availability of alternative fuels for consumers and the reliance of the residential sector on the fuel product to be banned [9] - **Diffusion** to other contexts: Due to the effectiveness of the coal ban, the intervention is now considered a good example for urban areas where heating with coal is standard practice[9] - **Sustainability**: the original ban from 1987 is still sustained in Dublin, although having been adapted [4] |
|  | Patient and organizational outcomes achievement |  | - Average black smoke concentrations in Dublin declined by 35.6 mg/m(3) (70%) after the ban on coal sales. Adjusted non-trauma death rates decreased by 5.7% (95% CI 4-7, p<0.0001), respiratory deaths by 15.5% (12-19, p<0.0001), and cardiovascular deaths by 10.3% (8-13, p<0.0001). Respiratory and cardiovascular standardised death rates fell coincident with the ban on coal sales. About 116 fewer respiratory deaths and 243 fewer cardiovascular deaths were seen per year in Dublin after the ban [26] |

1. Damschroder, L.J., et al., *Fostering implementation of health services research findings into practice: a consolidated framework for advancing implementation science.* Implement Sci, 2009. **4**: p. 50.

2. Stetler, C.B., et al., *A Guide for applying a revised version of the PARIHS framework for implementation.* Implement Sci, 2011. **6**(99).

3. 1990, A.P.A., *Air Pollution Act, 1987 (Marketing, Sale and Distribution of Fuels) Regulations, 1990*, in *S.I. No. 123/1990*. 1987.

4. Dockery, D.W., et al., *Effect of air pollution control on mortality and hospital admissions in Ireland.* Res Rep Health Eff Inst, 2013(176): p. 3-109.

5. Burns, J., et al., *Interventions to reduce particulate matter air pollution and their effect on health.* Cochrane Database of Systematic Reviews, 2014(1).

6. Air Quality Section, D.o.t.E., Community & Local Government. *Smoky coal ban*. 2016 [cited 2017 January 13]; Available from: <http://www.housing.gov.ie/environment/air-quality/coal/smoky-coal-ban>.

7. Damschroder, L.J., et al., *Fostering implementation of health services research findings into practice: a consolidated framework for advancing implementation science.* Implement Sci, 2009. **4**.

8. Kennedy, I., *Learning from Bristol: the report of the public inquiry into children’s heart surgery at the Bristol Royal Infirmary 1984–1995.* . Vol. CM 5207(1). 2001 London: The Stationery Office.

9. McLoughlin, E., *An Economic Analysis of Air Pollution Regulation: A Case Study of the Ban on Bituminous Coal*. 2001: Dublin.

10. Barry, A., *“It creeps menacingly”: When deadly smog choked Dublin’s skies*, in *TheJournal.ie*. 2013.

11. Sabatier, P.A., *Theories of the Policy Process*. 2007: Westview Press.

12. Lysdahl, K.B., et al., eds. *Guidance for assessing effectiveness, economic aspects, ethical aspects, socio-cultural aspects and*

*legal aspects in complex technologies*. 2016.

13. Young, M. *DUBLIN BAN ON SALES OF BITUMINOUS COAL*.

14. Kelly, O., *How the coal ban dealt with Dublin’s burning issue*, in *The Irish Times*. 2015: Dublin.

15. Rule, S., *Dublin Journal; Fair Is City but Foul Is Air When Smog Creeps In*, in *The New York Times*. 1989: Dublin.

16. European Network for Health Technology Assessment (EUnetHTA). *Glossary of HTA Adaptation Terms*. 2007 [cited 2013 June 26]; Available from: https://eunethta.fedimbo.belgium.be/sites/5026.fedimbo.belgium.be/files/Glossary%20of%20HTA%20Adaptation%20Terms.pdf.

17. Hogan, P., *Phil Hogan: I want smoky coal to be banned within the next 3 years*. 2013.

18. The Cochrane Collaboration. *Glossary*. c2004-2006 [cited 2013 03/05]; Available from: [www.cochrane.org](http://www.cochrane.org).

19. Union, I.G. *Case Studies in Improving Urban Air Quality*. 2016.

20. Rychetnik, L., et al., *Criteria for evaluating evidence on public health interventions.* J Epidemiol Community Health, 2002. **56**(2): p. 119-27.

21. Hage, E., et al., *Implementation factors and their effect on e-Health service adoption in rural communities: a systematic literature review.* BMC Health Serv Res, 2013. **13**(19).

22. Castro, F., M. Barrera, and C. Martinez, *The cultural adaptation of prevention interventions: resolving tensions between fidelity and fit.* Prev Sci, 2004. **5**(1): p. 41-5.

23. Redmond, D., et al., *Demographic Trends in Dublin*, D.C. Council, Editor. 2012: Dublin.

24. Nilsen, P., *Making sense of implementation theories, models and frameworks.* Implement Sci, 2015. **10**: p. 53.

25. May, C.R., et al., *Development of a theory of implementation and integration: Normalization Process Theory.* Implement Sci, 2009. **4**(29).

26. Clancy, L., et al., *Effect of air-pollution control on death rates in Dublin, Ireland: an intervention study.* Lancet, 2002. **360**(9341): p. 1210-4.

27. Bohan, C., *Environment Minister announces review of ban on smokey coal*, in *TheJournal.ie*. 2012: Dublin, Ireland.

28. Proctor, E.K., B.J. Powell, and J.C. McMillen, *Implementation strategies: recommendations for specifying and reporting.* Implement Sci, 2013. **8**(139).

29. Pfadenhauer, L.M., et al., *Context and implementation: A concept analysis towards conceptual maturity.* Zeitschrift für Evidenz, Fortbildung und Qualität im Gesundheitswesen, 2015(0).

30. Aarons, G.A., et al., *Collaboration, Negotiation, and Coalescence for Interagency-Collaborative Teams to Scale-Up Evidence-Based Practice.* Journal of Clinical Child & Adolescent Psychology, 2014.

31. Damschroder, L.J. and H.J. Hagedorn, *A Guiding Framework and Approach for Implementation Research in Substance Use Disorders Treatment.* Psychol Addict Behav, 2011. **25**(2): p. 194-205.

32. Council, D.C. *Fuel Regulations*. 2017 [cited 2017 January 11, 2017]; Available from: <http://www.dublincity.ie/main-menu-services-water-waste-and-environment-air-quality-monitoring-and-noise-control-air/fuel>.

33. Dublin City Council, et al., *Dublin Regional Air Quality Management Plan*. 2012.

34. Agency, E.P., *Environmental Protection Agency Submission on the Review of the Smoky Coal Regulations*. 2012, Environmental Protection Agency: Wexford, Ireland.

35. Bohan, C., *Phil Hogan: I want smoky coal to be banned within the next 3 years*, in *Thejournal.ie*. 2013: Dublin.
